# Wetland conversion to farmland in Bure and Womberma *Woredas*, Northwestern Ethiopia: Implications for sustainable land use

**DOI:** 10.1371/journal.pone.0352888

**Published:** 2026-07-02

**Authors:** Belachew Getnet Eneyew, Workiyie Worie Assefa

**Affiliations:** 1 Bahir Dar University, Faculty of Social Science and Blue Nile Water Institute, Bahir Dar, Ethiopia; 2 Bahir Dar University, College of Science, Department of Biology and Blue Nile Water Institute, Bahir Dar, Ethiopia; University of Illinois at Urbana-Champaign, UNITED STATES OF AMERICA

## Abstract

This study investigates the reasons why wetlands transformed to farmland. The data were garnered using Landsat, questionnaire surveys, and key informant interviews. Descriptive statistics were applied to analyze LULC change and the perceptions of wetland ecosystem services and attitudes towards wetland cultivation. An ordered probit model was applied to examine the influence of household and experts’ attributes on their attitudes towards wetland cultivation. The findings indicated that despite acknowledging the direct and indirect benefits of wetlands, the majority of households and experts/ department heads have preferred transforming wetlands into farmland. Household age, asset holding size (land and livestock) and livelihood strategies (crop farming and non-farm) as well as the institutional perspectives of experts and department heads have significantly influenced the desire to convert wetlands into agricultural land. Driven by the rising young households’ interest and local governments’ goal to create jobs have led to the distribution of wetlands to unemployed graduates and landless households. Consequently, significant (48%) areas of the wetlands have been transformed into farmlands over the past 36 years. It was found that the environmental soundness of wetlands cultivation has not been considered when converting them into farmland. Efforts should be exerted to expand employment opportunities to minimize the heavy reliance of rural people on wetland farming. Besides, it is imperative to execute agro-ecological evaluation prior to converting wetlands into farmland to ensure farming decisions are environmentally responsible.

## 1. Introduction

Wetlands are the vital landscapes of the earth’s surface. They covered an area of over 12 to –16 million km², which represents approximately 6% to 8% of the planet’s total land area [1]. The extent of wetland coverage varied across continents. Asia holds the largest share (32%) of wetlands and followed by North America (27%) [1]. African wetlands comprise about a tenth of the planet’s total wetland area, making the continent a major contributor to these crucial ecosystems [2,3]. Ethiopia is recognized as one of African countries which endowed with extensive and diverse wetland resources. Around 26,424 km^2^ or 2% of the country’s total land area is covered by wetlands [4].

Wetlands provide diverse benefits, from tangible resources like water, food, and building materials to intangible values such as flood control, climate regulation, and cultural significance. The ecosystem services delivered by wetlands vary across different spatial scales, from global to local [5]. At global level, they play an important role for climate change adaptation and support for biodiversity [6]. At the local level, they play a crucial role in sustaining the livelihoods of community and ensuring food security particularly in Africa including Ethiopia in addition to their crucial hydrological and ecological functions. The livelihoods of millions of people rely directly on the wetlands’ essential provisioning services [1].

Despite the essential services they provide, wetlands are declining at alarming rates faster than any other ecosystems [7]. According to Darrah et al. [8], the global natural wetlands’ area shrank by roughly 35% from 1970 to 2015. The disappearance of inland wetlands was higher in developing countries particularly in Asia and Africa [1]. Like many other African nations, Ethiopia has experienced significant wetland areas loss over the last two decades. Assefa and Eneyew [4] reported a 20% reduction in freshwater wetland coverage of the country from 2000 to 2020.

Numerous factors are driving the accelerated loss of wetland ecosystems. Agricultural expansion, urbanization, climate change and infrastructural development are widely documented as the major drivers of global wetlands’ depletion [8]. Ballut-Dajud et al. [9] found that agriculture is responsible for the largest portion (25%) of global wetland areas loss. Consistent with global patterns, agricultural encroachment is the major contributor to wetland loss in Africa including Ethiopia [9,10]. As documented by Assefa and Eneyew [4], the conversion of wetlands to farmland accounts for the vast majority of these ecosystems area in the country. The micro-level studies have also documented the substantial (20–90%) contribution of farmland expansion for the decline of wetlands in the past three decades [4,11–13].

Various studies [14–17] reported that the farming practices in the wetlands’ ecosystems are not environmental or eco-friendly. Consequently, their hydrological and ecological functions have adversely been affected. Prior studies have further demonstrated [18,19] how wetland farming negatively affected the vital provisioning services and cultural services. On the other hand, wetland degradation has severely jeopardized the agro-ecosystems by interrupting vital hydrological cycles, depleting soil moisture, eliminating the natural pest controls and pollination agents, which lead to less resilient farms and lower crops output [20,21].

Despite providing tangible and non-tangible benefits, various factors have driven the transformation of wetlands into farmland. Some studies [22] reported that perceiving wetlands as “wastelands” is the principal factor for their conversion. Others [23,24] reported that giving more priority to immediate benefits than the long-term value of wetland ecosystem services has contributed to wetlands’ cultivation. Some others [25,26] stated that escalating human-wildlife conflicts, caused by primate crops raiding and predators attack on livestock, are pushing the local farmers to convert wetlands into agricultural fields. Lack of comprehensive wetland policy frameworks has also been cited as the principal reason for their conversion into cultivated fields [27]. The aforementioned arguments revealed that the underlying causes for reclaiming wetland ecosystems for farming differ across various locations. This highlights the need for area-specific studies to validate existing research findings and identify uncover specific regional factors. Thus, this study assesses the extent of wetlands transformation to agricultural fields over the past 36 years and investigates the factors that causes for the conversion of the wetlands into the farmland in three wetlands of Bure and Womberma *Woredas*, Northwestern Ethiopia.

## 2. Theoretical argument on the overexploitation of natural resources

There is no simple answer for why wetland ecosystems are transformed into the farmland. Different hypotheses have been postulated on the causes of natural resource degradation including wetlands. However, the debates on the reasons why the natural resources including wetlands degradation are framed by three foundational perspectives: tragedy of the commons, Maslow’s Hierarchy of Needs and institutional theory.

The tragedy of the commons contends that the shared limited resources are depleted as a result of individuals’ actions driven independently by their own self-interests without considering the future sustainability of the natural resource. In other words, the common property resources are subject to overexploitation as a result of the absence of clear-cut and strictly followed rules of engagement, which usually results in natural resource degradation. Wetlands are among the natural resources which commonly regarded as “the common property resources” [28].

Maslow’s Hierarchy of Needs theory, on the other hand, argues that the people who are struggling at the edge of subsistence levels of consumption are preoccupied with surviving in the present [29,30]. Thus, the use of the natural resource including wetlands is determined by immediate needs rather than the long-term sustainability of the wetland ecosystems, which potentially leads to wetland’ degradation [31].

Institutional theory stands in opposition to tragedy of the commons and Maslow’s Hierarchy of needs. This theory challenges the fundamental assumption that natural resources depletion is driven by self-interested actors (tragedy of the commons) and survival-driven behavior (Maslow’s Hierarchy of needs) [32,33]. According to this theory, resource degradation stems from institutional failures (policies, laws, and regulations; organizations; norms, traditions, practices, and customs). It argues that lack of or poorly designed policies, laws and regulations coupled with ineffective organizational practices contribute to overutilization of natural resources [33].

Empirical studies have been conducted to validate whether or not the aforementioned hypothetical factors contributed to natural resource degradation. The findings of these studies showed contradictory results. The studies that examined [34–36] the link between property regimes and wetland degradation has challenged the “Tragedy of the Commons” hypothesis. As per the findings of these studies, the communal wetlands are better managed than the privatized wetlands. [34] and [36] observed that privatization of wetlands has a more significant contribution to ecological and hydrological alterations than when wetlands are managed as common-pool resources. The hypothetical factors, which are assumed to be the causes for natural resource degradation by institutional theory, were validated by various studies [4,37,38].

Even though some studies [39,40] have questioned Maslow’s theory of immediate needs fulfillment, others confirmed the contribution of prioritizing the short-term gains from natural resources over long-term ones to resource degradation [41,42]. Specifically, the study done by [43] in a wetland ecosystem highlights how the immediate needs of buffer zone communities can negatively affect the wetland’s vital services, thereby aggravating the degradation of the wetland ecosystem.

In this study, it was hypothesized that the inefficiency of government institutions in creating non-farm employment opportunities alongside problematic land tenure policies and other institutional issues are significant drivers of wetland cultivation in the study area. These factors create pressure on land resources, pushing the local administrations to consider allocating the wetlands for unemployed college and university graduates for agricultural purposes. Young farm households, particularly those lacking access to land through inheritance or family gifts, combined with limited opportunities for alternative livelihoods are expected to put considerable pressure on local administrations to distribute wetlands for farmland. The allocation of wetlands for cultivated land would not expect to be driven by local officials and farmers understanding of the wetlands’ tangible and non-tangible benefits.

## 3. Research methods

### 3.1. Description of the study wetlands

**Fig 1:**
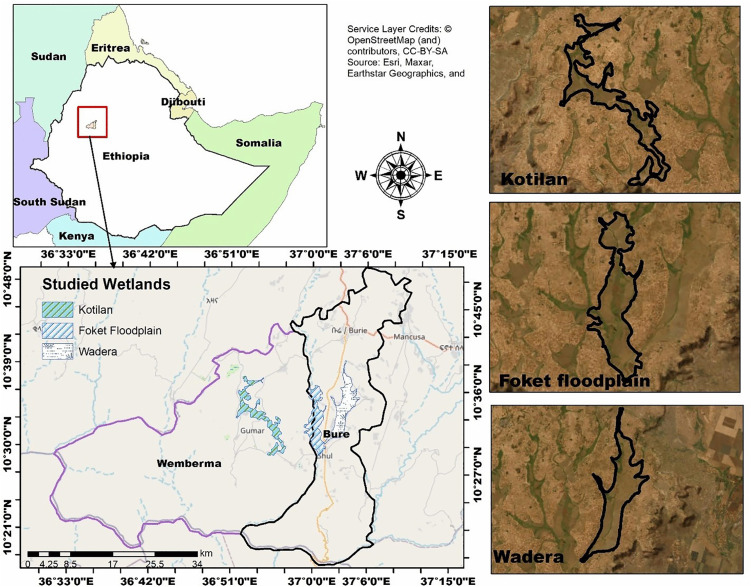
Location map of the study wetlands. Source attribution: Service Layer Credits: ©. OpenStreetMap (and) contributors, CC-BY-SA; Source: Esri, Maxar, Earthstar Geographics, and the GIS User Community.

This study was conducted in three wetlands (Kotlan, Foket, and Wadera) that are located in the Fetam River watershed (Fig 1). Administratively, they are located in Bure and Womberma *Woredas* in Amhara Region, northwestern Ethiopia. The sizes of these wetlands ranges from 1,564 ha to 1,703 hectares. Based on records from 1981 to 2020, the monthly mean rainfall ranges from 6.4 to 344.1 mm, and the mean monthly temperature varies between 17.9 and 22.7 °C with a mean temperature of 19.8 °C. The average annual precipitation in the watershed is 122.1 mm. The highest rainfall is recorded from June to September, and the lowest from February to May

Central Statistical Service [44] projected that the total population of Bure and Womberma Woreda in 2021 was 171,792 and 126,242, respectively. Of these, 68,209 people were inhabitants of the *kebele* administrations which are adjacent to the study wetlands. The primary economic activity of the people is a subsistence mixed crop-livestock farming system. Cereal crops such as maize and wheat are dominant crops. Red pepper is also widely grown by the large majority of farmers in the two *woredas*. As reported by [45] and [46], these two *woredas* were major producers of maize, wheat, and red pepper in Ethiopia. Besides, livestock husbandry (cattle, sheep, goats, donkeys and mule) makes a significant contribution to the livelihood of the farmers. Based on the agricultural offices of the two *woredas*, 67095.3 TLU were kept in the *kebele* administrations which are adjacent to the wetlands [11]. Keeping such larger livestock population in conjunction with crop production may create heavy pressure on the study wetlands.

The wetlands under study are located within river channels, making them riverine. Surface water during rainy season and groundwater are the main water sources for these wetlands. Their water levels are typically high between July and December, and many of their parts recede from January to June.

### 3.2. Methods of data collection

#### 3.2.1. Landsat images.

The first step of this study was investigating the land use and land cover dynamics in the wetlands to see the extent of wetlands’ cultivation, and then to investigate why wetlands have been cultivated in the study areas. We used Landsat image series, including Landsat 5 Thematic Mapper (TM), Landsat 7 Enhanced Thematic Mapper Plus (ETM+), and Landsat 8 Operational Land Imager (OLI/TIRS) for the years 1985, 1995, 2010, and 2021 (see Table 1). The quality of the Satellite image compared to the previous periods, government policy intervention, and land redistribution were the criteria for choosing 1985, 1995, 2010 and 2021, respectively.

**Table 1:** Characteristics of satellite images used to analyze LULC of wetlands.

Year acquired	Satellite	Sensor	Spatial resolution	Path/Row	Bands used & its range of wavelengths	
					Bands	Wave length
1985, 1995, 2010	Landsat 5	MSS/TM	30m × 30m	170/53	1, 2, 3, 4, 5 & 7	0.45-2.35µm
2000	Landsat 7	ETM+	30m × 30m	170/53	1, 2, 3, 4, 5 & 7	0.45-2.35µm
2021	Landsat 8	OLIS/TIRS	30m × 30m	170/53	2, 3, 4, 5, 6 & 7	0.45-2.25µm

The 1985, 1995, and 2010 MSS/TM (path/row 170 and 53) and the 2021 OLIS/TIRS (path/row 179 and 53) images were obtained from the Google Earth Engine (GEE) platform via code.earthengine.google.com. The shapefiles for the studied wetlands were digitized using Google Earth Pro and subsequently converted from KML format to ESRI shapefiles within the ArcGIS platform. This digitization process did not include all wetland parcels within their respective micro-watersheds, particularly the Kotlan site (only the downstream portion was delineated in the final shapefile representation).

All datasets are Level 2, Collection 2, Tier 1, which have undergone geometric, radiometric, and atmospheric corrections by the image provider (U.S. Geological Survey) and are thus considered the highest quality. From these collections, we selected Bands 1–7 (excluding Band 6) from Landsat 5 and Landsat 7, and Bands 2–7 from Landsat 8. Only images with less than 10% cloud cover were composited in GEE for each year from January 1 to December 30, with the median value applied based on the geometry of the study region.

The stacked images were then imported into ArcGIS 10.8 and clipped to the respective wetland areas using their shapefiles. The clipped images were processed and analyzed using various band combinations, such as true-color composite and false-color composite, to facilitate land cover classification. The classification procedure involved the following steps. First, we acquired 50–60 training samples for each land cover class, Second, we created signature files by combining the stacked images with the training samples and finally, the images classified into their respective land cover classes through maximum likelihood supervised classification by using the signature files. The identified land cover classes included water, dense vegetation, low vegetation; farmland covered by crop residue, and plowed farmland. The classification results were then reclassified into wetland and cultivated land using spatial analysis tools in ArcGIS 10.8.

During the digitization of training samples, careful consideration was given to selecting representative sites with sufficient pixel coverage, since the wetland areas were of manageable size. High-resolution imagery from Google Earth Pro was consulted to ensure accuracy, and the classification results demonstrated excellent accuracy.

Accuracy assessment: The overall accuracy and a Kappa analysis (equation 1) were performed to validate each land cover class of the study periods (1985, 1995, 2000, 2010 and 2021) based on the confusion matrix. The confusion matrix shows the user’s accuracy, the producer’s accuracy, and the Kappa coefficient. Users’ accuracy is the probability that the land cover classes identified on the map is classified into the same category on the ground, whereas producer’s accuracy is the probability that the land covers identified on the ground is classified into the same category on the map (Lillesand et al., 2015). The confusion matrix compared each pixel in the reference map with its corresponding pixel in the classified map.


$$\begin{array}{c} Kappa=\frac{observed\;accuracy-chance\;of\;agreement}{\text{1}-chance\;of\;agreement} \end{array}$$

The confusion matrix reveals that an overall accuracy of LULC for four reference years (1985, 1995, 2000 and 2010) was 95.50% while it was 97.27% for 2021. The Kappa coefficient of the LULC for the five reference years were 78.15, 79.93, 81.72, 88.96 and 87.19%, respectively. These indicate strong agreement between the classification and reference data. High classification accuracy was observed across both LULCs, with user’s accuracy of the reference years ranging from 86.67% to 96.66% and producer’s accuracy spanning from 86.79% to 94.92%, demonstrating strong reliability in identifying wetlands and cultivated land.

#### 3.2.2. Survey questionnaire.

A household survey was undertaken from the second week of March to the end of May 2022 for getting valuable insights about the perception of rural households on the wetlands’ ecosystem services, their attitudes towards the wetlands’ cultivation, and analyzing the implications of household characteristics on wetland cultivation. A sample of 396 households, drawn from 17 villages bordering wetlands, participated in the survey. Purposive and systematic sampling techniques were applied to select the sample villages and households, respectively. The sample households were proportionally divided among the seventeen sampled villages to obtain the sample size for each village.

A questionnaire survey was also undertaken with 75 experts and heads of two *woredas* working in offices of agriculture, environmental protection, land use and administration, irrigation development and livestock and fishery resource development. The aims of experts/heads of department survey were to: (1) investigate how the personal profiles and the missions of sector offices affected experts/heads’ attitudes towards wetlands cultivation and (2) analyze the implications of experts/heads attitudes on the conversion of wetlands to cultivated fields.

Demographic profiles, awareness of wetland ecosystem services, and opinions on wetlands cultivation were included in both questionnaires prepared for households and experts surveys. The question items incorporated under household profile section consists of age, sex, marital status, education background, family size and livelihood activities. Age, marital status, sex and educational background were also included under personal profile section of expert’s questionnaire in addition to their working experience, position held, and field of specialization. The question items in the wetland ecosystem perception sections for both households and experts’ questionnaires covered the importance of wetlands in the delivery of provisioning, regulating, supportive and cultural services (see S1 Table). The attitude section of the questionnaires for both experts and households incorporated the items concerning opinions on wetland cultivation and sustainable management. Likert-scale type questions were prepared to elicit households’ and experts’/department heads’ perceptions of wetland ecosystems and their attitudes toward wetland cultivation.

Both household and expert questionnaires were written in English and then translated into the local language (Amharic) to ensure ease of communication with the respondents. Household questionnaires were pre-tested on 25 respondents to confirm that the questions were unambiguous. The household survey was administered through face-to-face interviews by 13 enumerators, whereas the experts/heads of offices survey was self-administered.

#### 3.2.3. Key Informant Interviews (KIIs).

KIIs were held with 12 persons to cross-validate the data acquired from Landsat Images and gather insights regarding the factors leading to the transformation of wetlands into cultivated fields. The key informants were (i) elders with ages above 70 years and had indigenous knowledge; (ii) former *kebele* administration (KAs) members who participated in the 1997 land redistribution, and (iii) four KAs chief administrators. At the beginning of each KII, the purpose of the study was explained, and prior verbal consent was obtained. Interviews were audio-recorded with key informants’ consent, and notes were taken at the same time to capture important cues and relevant points

### 3.3. Methods of data analysis

Various approaches were employed to analyze the data collected through multiple sources. Thematic analysis was applied to analyze the data collected through KIIs. The analysis started by transcribing key informant interviews. The transcribed data were then categorized into themes and finally converted to generate the smallest meaningful units. Descriptive statistics were used to analyze the demographic and socioeconomic characteristics of the respondent households and experts, the perception of respondents on wetland ecosystem services, their attitude towards wetland cultivation. Statistical Packages for Social Sciences (SPSS) version 26 was used for the analysis of descriptive statistics.

An ordered probit regression model was adopted to analyze the factors influencing the attitudes of households and experts towards wetlands conversion. The regression analysis was performed using STATA software (version 18.0).

#### 3.3.1. Model selection and specification.

The fundamental justification for selecting an ordered probit model rests upon the ordinal nature of dependent variable (attitude towards wetland cultivation). The attitudes of households and experts towards wetland cultivation are classified into five ordinal categories (1=  strongly disagree, 2 = disagree, 3 =  No opinion, 4 =  agree and 5 =  strongly agree) [47–49]. Ordered probit model measures the probability that the dependent variable ($Y_{i}$, for the i^th^ mean scale) falls in one of the discrete categories conditioned on levels of the independent variables ($X_{j}$). Suppose the households and experts’ attitude i ($Y_{i}$) is the unobserved variable (latent variable) and is expressed in the following equations ($Q_{\text{1}}$ and$Q_{\text{2}}$)


$$\begin{array}{cc} HHAT= & \beta_{\text{0}}+\beta_{\text{1}}*HHG+\beta_{\text{2}}*MHH+\beta_{\text{3}}*\text{HHAG}+\beta_{\text{4}}*FSIZ+\beta_{\text{5}}*EDUCH\\ & +\beta_{\text{6}}*\text{CRENG}+\beta_{\text{7}}*OFFEN+\beta_{\text{8}}*FAROWN+\beta_{\text{9}}*LASIZ+\beta_{\text{1}\text{0}}*LIVEOWN\\ & +\;\beta_{\text{1}\text{1}}*LIVESIZ+\beta_{\text{1}\text{2}}*MEANPERC+£\ldots \ldots \ldots \ldots \ldots \ldots \ldots \ldots \ldots \ldots Q_{\text{1}} \end{array}$$

Where HHAT= household respondent attitude towards wetland cultivation, $\beta_{\text{0}}$ = is a constant, $\beta_{i}$ is representing the coefficient of independent variables described in Table 2, and ε is the error term.

**Table 2:** The description of household variables used in ordered probit model estimation.

Predictors	Description of variables	Hypothesized relationship
Household attitude (HHAT)	The attitude of the household head towards wetland cultivation (categorical variable, the value ranged from 1 to 5)	
Independent Variables		
Gender of household head (HHG)	Dummy = 1 if the household head is male = 0 female	Positive
Marital status (MHH)	Dummy variable = 1 if the household head is married = 0 otherwise	Positive
Age of household head (HHAG)	Continuous variable = the age of household heads in years	Negative
Family size (FSIZ)	Continuous (The number of family members in the household)	Positive
Educational status (EDUCH)	Continuous variable (from 0 which represent unable to read to grade 12)	Negative
Engagement in crop production (CRENG)	Dummy variable = 1 if the household engaged in the crop production = 0 other wise	Positive
Engagement in off/non-farm activities (OFFEN)	Dummy variable = 1 if the household engaged in off or non-farm activities = 0 otherwise	Negative
Farmland ownership (FAROWN)	Dummy variable = 1 if the household head own farm land = 0 otherwise	Negative
Landholding size (LASIZ)	Continuous variable; the farmland size owned by the household in hectare	Negative
Livestock ownership (LIVEOWN)	Dummy variable = 1 if the household own livestock = 0 otherwise	Negative
Livestock holding size (LIVSIZ)	Number livestock owned by the household measured in tropical livestock unit (TLU)	Negative
Perception of respondents (MEAPERC)	Aggregated mean perception of the household head on the wetland ecosystem	Negative


$$\begin{array}{cc} HHAT= & \beta_{\text{0}}+\beta_{\text{1}}*EXSEX+\beta_{\text{2}}*AGEXP+\beta_{\text{3}}*+\beta_{\text{4}}*FSIZ+\beta_{\text{5}}*EDUEXP+\beta_{\text{6}}*\text{EXPY}\\ & +\beta_{\text{7}}*AGRIO+\beta_{\text{8}}*LFDO+\beta_{\text{9}}*LUAO+\beta_{\text{1}\text{0}}*ENVPRO+\beta_{\text{1}\text{1}}*MEANPERC\\ & +£\ldots \ldots \ldots \ldots \ldots \ldots \ldots \ldots \ldots \ldots \ldots \ldots \ldots \ldots \ldots \ldots \ldots \ldots \ldots Q_{\text{2}} \end{array}$$

Where, EXAT= experts’ attitudes; β_0_ is a constant, β_i_ is the coefficient of independent variables described in Table 3, and ε is the error term.

**Table 3:** The description experts/department heads variables used in the order probit model estimation.

Variables	Description of variables	Relationship
EXAT	Attitude towards wetland cultivation (categorical variable, the value ranged from 1 to 5)	
EXSEX	Sex of experts and department heads (Dummy = 1 if an expert is male = 0 otherwise)	Positive
AGEXP	Age of experts/ department heads (Continuous variable)	Negative
EDUEXP	Educational status of experts/ department heads (Continuous variable; years of schooling)	Negative
EXPY	Years of experience (Continuous variable)	Negative
AGRIO	Working in an agricultural office (Dummy variable = 1 if an expert is working in an agriculture office = 0 otherwise)	Positive
LFDO	Working in livestock and fisheries development (Dummy variable = 1 if an expert is working in livestock dev’t office = 0 otherwise)	Negative
LUAO	Working in land use and land administration office (Dummy variable = 1 if an expert is working in land administration office = 0 otherwise	Positive
ENVPRO	Working in environmental protection office (Dummy variable = 1 if an expert is working in environmental protection office = 0 otherwise)	Negative
IRDEVO	Working in irrigation development office (Dummy variable = 1 if an expert is working in irrigation development office = 0 otherwise)	Positive
MEANPERC	Aggregate mean perception of experts on wetland ecosystem (Categorical variable; the value ranged from 1 to 5)	Negative

#### 3.3.2. Descriptions of Independent variables.

Households’ variables: As reported by previous studies [48,49], gender, age, marital status, educational level, family size, land ownership, the size of farmland, livestock holding size in tropical livestock unit (TLU), and perception on wetland ecosystem services determined local people attitudes towards wetland management. This study also expected the influence of these variables on the households’ attitudes towards wetlands’ cultivation. Most of these independent variables were expected to have a negative relationship with dependent variables, except gender and engagement in crop production. The descriptions of household variables are summarized in Table 2.

Experts and departments’ variables: It was also assumed that the attitudes of experts and department heads were determined by the sector offices that employed them. Experts working in agriculture, land use and administration, and irrigation development offices are expected to support the allocation of wetlands for farmland, assuming that the missions of these sector offices are to ensure household food security at any environmental cost. As stated by the rural development strategy document of Amhara Region (2007), no parcel of land would be left fallow, and every droplet of water resource would be used for crop production. This clearly indicates that agriculture, land use, and administration and irrigation development offices have given more priority to food production for food self-sufficiency than to sustainable wetland management. In addition, wetlands have been perceived by the government as a major source of irrigation water for the expansion of irrigated agriculture. On the contrary, the mission of the environmental protection office is to promote and ensure the protection of the wetland ecosystem [37]. Similarly, the livestock development and fishery resource development office has a strong interest for the conservation of wetlands because of viewing them as the source of livestock feed and the production of fish. Thus, it was expected that the experts/department heads working in these two offices would strongly disagree or disagree on the cultivation of wetlands. Besides, the experts/ department heads’ attitudes towards wetland cultivation are assumed to be influenced by their sex, education status, age, working experiences, and the aggregated mean perception on the wetland benefits [50,51]. It is assumed that experience, education, and age of experts would negatively influence the attitude of experts towards wetland change.

### 3.4. Ethical approval

The ethical aspects of this research proposal were evaluated and approved by the review committees of the Blue Nile Water Institute and the Research and Community Engagement vice Office in accordance with Bahir Dar University’s 2022 research guideline. After the approval of the review committees, a letter requesting support was written from the office of the University Research and Community Engagement Vice President, and then the permission letters were obtained from the study *woreda* administrations.

After obtaining permission from the *woredas*’ sector offices, research participants were fully informed about the purpose of the research and how the data obtained from them would be used. Informed consents were then requested from respondents, key informants, and FGD participants. Household survey participants voluntarily agreed to participate and provided informed consent by signing the consent form provided on the first page of the questionnaire. The key informants and the FGD participants provided their unwritten, free assent to participate, having demonstrated an understanding of the study’s implications and their right to refuse. Verbal agreements obtained from them are documented using a digital voice recorder. The completed questionnaires and audio recordings were submitted to the institutional review board, and then the IRB confirmed proper participant consent and agreements were secured.

## 4. Results

### 4.1. Land use land cover changes of the study wetlands

**Fig 2:**
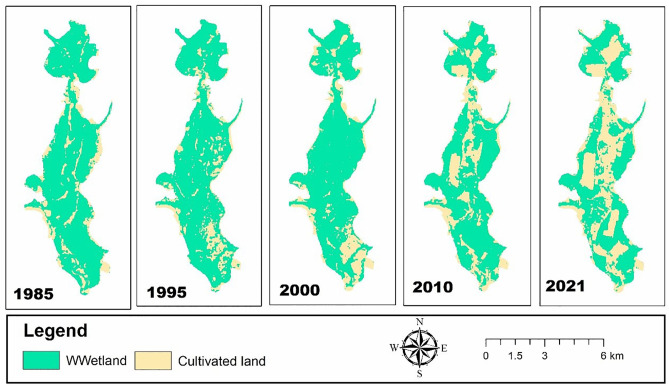
Spatiotemporal cover change of the Foket floodplain wetland. Data source: Landsat TM (1985 & 1995), Landsat 7 ETM+ (2000 & 2010), and Landsat-8 OLI (2021) images courtesy of the US Geological Survey.

**Fig 3:**
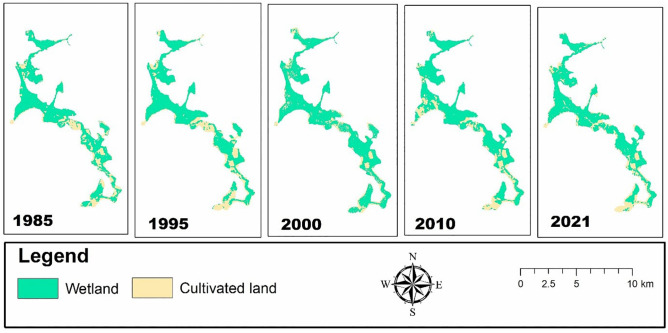
Spatiotemporal cover change of the Kotlan wetland, specifically in its downstream area. Data source: Landsat TM (1985 & 1995), Landsat 7 ETM+ (2000 & 2010), and Landsat-8 OLI (2021) images courtesy of the US Geological Survey.

**Fig 4:**
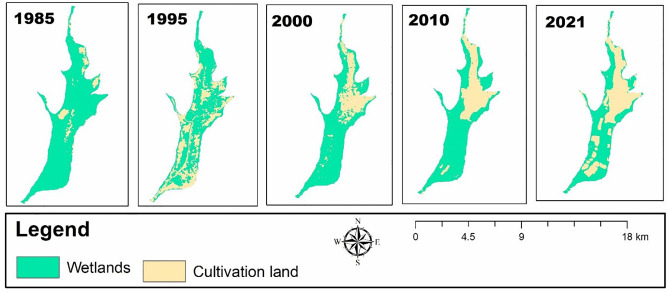
Spatiotemporal cover change of Wadera Wetland. Data source: Landsat TM (1985 & 1995), Landsat 7 ETM+ (2000 & 2010), and Landsat-8 OLI (2021) images courtesy of the US Geological Survey.

The LULC changes for the study wetlands spanning from 1985 to 2021 are shown in Table 4, Figs 2–4. As indicated in Table 4, in 1986, the Kotlan, Foket, and Wadera areas were predominantly wetland, with 93%, 81.8%, and 91.8% coverage, respectively. By 2021, these percentages had decreased to 72.8%, 56.1%, and 54.1%, respectively. This clearly indicated that 3355 ha of wetlands’ areas have been converted to farmland over the past 36 years. In terms of percentage, the total area of converted farmland within the study period was increased by 48.8% in 2021. A higher conversion of wetlands was observed in the second study period (1995–2000) may be due to the allocation of wetlands during the 1997 land redistribution, followed by the last study period (2010–2021). The extent of wetland conversion to farmland varied across three study wetlands from 34.8% in the Kotlan Wetland to 69.7% in Wadera Wetland.

**Table 4:** The LULC changes of the three wetlands from 1985 to 2021.

Wetlands name	LULC	Years					% change				
		1985	1995	2000	2010	2021	1995−1985	2000−1985	2010−2000	2021−2010	2021−1985
Kotlan	Remained Wetland	2417	2378	2076	1955	1793	−1.6	−12.7	−5.8	−8.3	−34.8
	Converted to farmland	183	222	524	645	807	21.3	136.0	23.1	25.1	77.3
	Total	2600	2600	2600	2600	2600	0.0	0.0	0.0	0.0	0.0
Foket	Remained Wetland	2381	2294	2215	1949	1564	−3.7	−3.4	−12.0	−19.8	−52.2
	Converted to farmland	407	494	573	839	1224	21.4	16.0	46.4	45.9	66.7
	Total	2788	2788	2788	2788	2788	0.0	0.0	0.0	0.0	0.0
Wadera	Remained Wetland	2809	2411	1936	1787	1655	−14.2	−19.7	−7.7	−7.4	−69.7
	Converted to farmland	250	648	1223	1272	1404	159.2	88.7	4.0	10.4	82.2
	Total	3059	3059	3059	3059	3059	0.0	0.0	0.0	0.0	0.0
All	Remained Wetland	7607	7083	6127	5691	5112	−6.9	−13.5	−7.1	−10.2	−48.8
	Converted to farmland	840	1364	2320	2756	3435	62.4	70.1	18.8	24.6	75.5
	Total	8447	8447	8447	8447	8447					

The qualitative data confirmed that parts of wetlands have long been cultivated after their water receded. The size of the cultivated wetland plots, however, varied from period to period. As per elders’ informants, the amount of farmland within the wetlands and its peripheries before the early 1980s was insignificant. In the 1980s, wetland cultivation had expanded from their edges to the main parts due to the allotment of wetlands to newly formed households by the local government.

The wetlands’ cultivation was exacerbated during the transition period (1991–1994). The informants reported that large parts of the study wetlands were cultivated in the first three consecutive years after the Ethiopian People’s Revolutionary Democratic Front (EPRDF) captured the reins of the state. The breakdown of state control was the principal factor for their widespread farming during the transition period. It was reported during the interview that the farmers with legally owned land in peripheries and farmers without landholding participated in the cultivation of wetlands during the transition period. The wetlands’ parts that were farmed during the transition period were reclaimed four years later owing to emphasis placed on the natural resource conservation including wetlands.

The restoration and conservation of the study wetlands, however, has not been sustained for several years. It was noted from KIIs and FGDs that the wetlands’ cultivation has been widely transformed into farmland since 1997 land redistribution. One of the land redistribution committee members of KAs around the Wadera wetland described the allocation of the parts of the wetland during the 1997 redistribution as:

*In our kebele administration, farmers who were at least 18 years old were registered to get farm land. There was a gap between the numbers of applicants compared to the registered land area. First, we redistributed long-time cultivated lands to the existing household and then we distributed 150 hectares of the Wadera wetland to 300 people, whose age were 18 years old and did not get land*.

The allocation of wetlands to the young households had been continued since the late 2000s. The chief administrator of the KA located adjacent to the Foket floodplain, reported that:

*We distributed 250 hectares of the flood plain area to 900 people in 2010. These people were young landless households and unemployed TVET graduates who had claimed land redistribution since the millennium.*

Similar actions had been taken in Kotlan and Wadera Wetlands. As reported by the KA administrations, 384.9 hectares of these wetlands were distributed for farmland to 1449 landless households and unemployed youths from 2016 to 2020. Records from the small-scale enterprise and job creation offices revealed that the local administrations intend to distribute the remaining wetlands to 6,225 unemployed TVET and university graduates by organizing them into different working groups.

The transformation of wetlands into farmland has profound negative implications for flora and fauna. During interviews, elder informants witnessed the migration and loss of fauna driven by the conversation of wetlands into farmlands. According to them, the macrophytes and woody plants were dense prior to three decades ago. They harbored antelopes, monkeys, various bird species, etc. As per KIIs, the density of macrophytes and woody plants, however, has been decreased and consequently, a corresponding reduction in mammalian, reptiles and avian populations was observed. The wetlands’ cultivation has also negative implication on the hydrological functioning of wetlands. During a field survey, it was noted that the diversion of wetlands’ water for irrigation has resulted in the reduction of water volume particularly during dry seasons.

### 4.2. The survey participants’ profiles

The demographic profile of household respondents illustrated that male respondents constituted 92.5% of the total, with females comprising the remaining 8.5%. Close to 90% of them were married, with the remaining (11.5%) comprised single, divorced or widowed. The age of household heads averaged 46 years, ranging between a minimum of 23 and a maximum of 83 year. About 60% of the respondents were under the age group of 23–50 years. They had an average family size of 5.2 persons. More than half of the households had a family size of more than 5 people, which suggested that they need more resources to survive and puts pressure on the wetland. The mean (2.8) educational level of the respondents indicates that the level of education of the majority of the respondents was adult education, and primary education. Lower levels of formal education are often linked to a lack of awareness of the crucial ecological functions of wetlands [52]. The cultivation of food crops was the fundamental means of employment for the entire surveyed households. Reliant on crop production place high demand on farmland resulting in increased pressure on the wetlands [53]. Nine in ten households reared livestock with a mean size of 4.6 heads. Nearly 90% of them owned cultivated land with an average landholding size of 1.27 hectares. Only 1/5th of them engaged in off-and non-farm livelihood options.

The demographic profile of experts and department heads revealed that 88% were male, with the majority employed in the Office of Agriculture (45%) and the Land Use and Administration Office (24%). The mean age of respondents was 34 years, but ranged from 22 to 56 years. The mean of educational status suggests that most of them graduated with first degree, and their average working experience was 10.44 years. The higher educational background and relatively long working experience reflect a better recognition of various ecosystem services provided by wetlands (Nyongessa et al, 2016)

### 4.3. Perception of households on wetland ecosystem services

**Fig 5:**
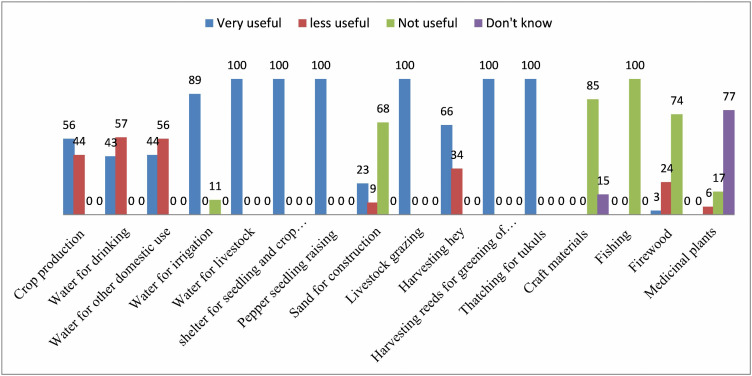
The perception of farmers on the provisioning services delivered by the study wetlands.

The households’ perceptions on the various wetlands’ provisioning services are summarized in Fig 5 and S2 Table. As illustrated in the figure, all survey participants acknowledged the vital role of wetlands for livestock watering, seedlings and crop residue shelter, seedling raising, fodder grazing, floor greening (*cheffee*), and thatching grasses. Nearly 90% and almost two-thirds of them have also recognized the vital contribution of these wetlands to irrigation agriculture and hay harvesting, respectively (Fig 5). Besides, the percentage of respondents who perceived the essential value of the wetlands to recession farming (55.9) and human drinking water supply (45%) was significant (see the source data, S3 Table).

**Fig 6:**
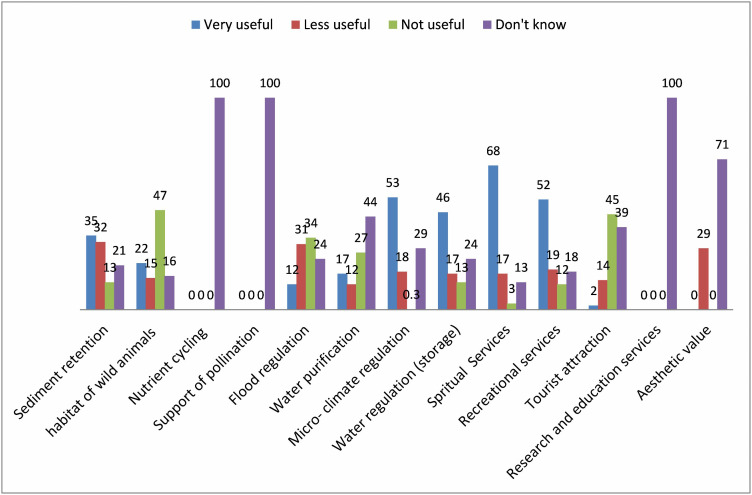
The perception of respondent households on regulating, supporting, and culturalservices delivered by the study wetlands.

The role of the study wetlands in providing supporting, regulating and cultural services have been recognized by the household participants as well. Sediment retention and wildlife habitat are among the supporting services acknowledged by more than one-third and a quarter of survey participants, respectively. A substantial portion of the surveyed households recognized water regulation (45.6%) and micro-climate regulation (32.9%) as key regulating services. Fig 6 also indicated that 76.6% and 51.9% of the survey participants identified spiritual and recreational values, respectively as the crucial cultural services provided by these wetlands.

### 4.4. Perception of experts on wetland ecosystem services

**Fig 7:**
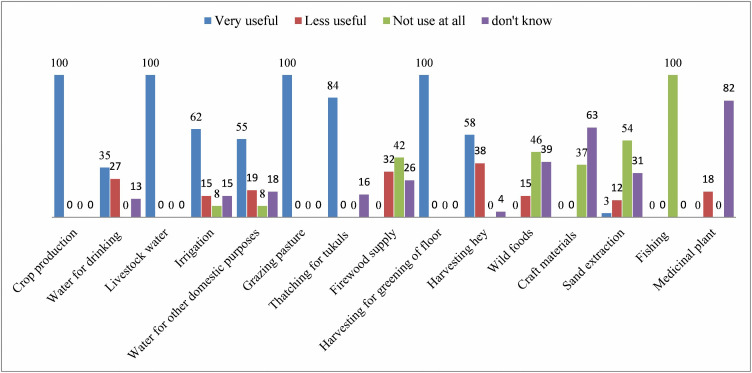
Perception of experts on the provisioning services provided by the study wetlands.

Like household respondents, experts and department heads have acknowledged the vital contribution of wetlands to provisioning services delivery. The important provisioning services recognized by all of surveyed experts/ department heads included crop production, livestock watering, livestock grazing, and reed harvesting for floor greening (*cheffee)*. The importance of wetlands for thatch grass harvest, irrigation farming, hay production, and domestic water supply was also known by 83.8, 62.1, 58.1, and 55.4% of experts, respectively (see Fig 7 and S3 Table).

**Fig 8:**
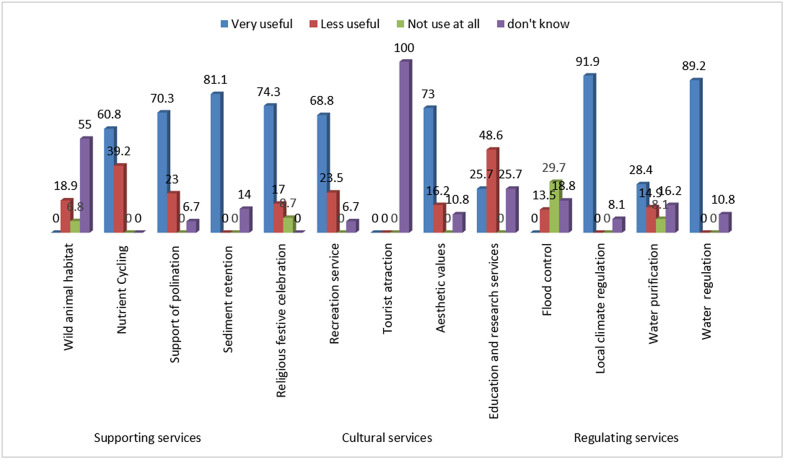
The perception of experts on the supporting, regulating and cultural services provided by the study wetlands.

Experts and departments’ heads also had a better understanding on the supporting, regulating, and cultural services delivered by the study wetlands’ ecosystems. The survey results summarized in Fig 8 illustrate that the majority of experts and department heads acknowledged the significant wetlands’ contributions of sediment retention (81%), pollination support (70.3%) and soil nutrient cycling (60.8%). The wetlands’ contribution to local climate regulation, water regulation (storage), and water purification was also recognized by 91.9%, 89.2% and 60.8% of experts/department heads, respectively. Furthermore, Close to 3/4th, 73%, 69.8% and 48.6% of experts observed the vital contribution of the wetlands to spiritual, aesthetic values, recreational services, educational and research services, respectively, as well (see the source data, S5 Table).

### 4.5. The attitudes of households towards wetland management

Despite recognizing the vital contribution of wetlands in providing ecosystem services, more than two-third of household respondents were not interested in managing or conserving wetlands. The regulated utilization of wetland resources was not also acceptable for more than 3/4th of households. More than half of them preferred the wetlands conversion to farmland instead of using them for other benefits. The principal driver behind the wetlands’-to-farmland conversion desired by the majority of respondents was not the eradication of the natural nesting grounds insects, reptiles and mammals, but rather the pressing need to address the shortage of farmland (see Table 5).

**Table 5:** The attitudes of the farmers towards the four wetlands in Bure and Womberma *Woreda.*

Question items	Strongly disagree	disagree	don’t know	Agree	Strongly Agree	Mean	S. D
Wetlands should be protected	8.1	59.4	0.0	32.0	0.6	2.6	1.04
The utilization of wetland resources should be regulated	16.7	62.2	0.6	19.0	1.4	2.3	0.99
Wetlands should be converted to farmland to remove the breeding ground of mosquitoes	26.2	55.3	6.3	11.8	0.3	1.9	0.74
Wetlands should be destroyed to eliminate the habitats of jackals	28.0	61.7	8.1	1.4	0.9	1.9	0.69
Wetlands should be destroyed to reduce snakes and other poisonous insects attacks	25.1	63.7	8.1	1.2	2.0	1.9	0.73
I prefer to use wetlands for agriculture rather than using them for other purposes	10.7	30.3	2.0	28.2	28.8	3.3	1.44
Wetlands should be destroyed to reduce the damage of crops by birds and primates	23.9	56.5	17.9	0.9	0.9	2.0	0.73
Wetlands should be changed to farmland to minimize the scarcity of farmland	7.5	28.4	1.7	34.6	27.7	3.3	1.33

### 4.6. Attitudes of experts towards wetland management

Similar to the farmers, the conservation or management of wetlands was not acceptable by the majority (71.6%) of the two *woredas*’ experts. As it is shown in Table 2, about 71.7% of the respondents supported the wetland conversion to farmland, and nearly 2/3rd of them perceived the wetlands allotment to landless households as the only option to address rural unemployment. More than half of them also promoted the diversion of wetlands’ water for small-scale agriculture (see Table 6).

**Table 6:** The attitudes of experts towards the conversion of wetlands into cultivated land in Bure and Womberma *Woreda.*

Question items	Strongly disagree	disagree	Don’t know	Agree	Strongly agree	Mean	S.D
Wetlands should be cultivated due to the shortage of farmland	6.8	9.5	12.2	27.0	44.6	3.9	1.25
The government should take actions to protect wetlands	12.2	39.2	14.9	21.6	12.1	2.8	1.25
Wetland water should be diverted for irrigation	9.5	20.3	17.6	39.2	13.5	3.3	1.21
Wetland cultivation is the only option to address rural unemployment	9.5	14.9	9.5	43.2	23.0	3.6	1.26

### 4.7. The influence of institutional and personal profiles of farm households and experts on their attitudes towards wetland cultivation

The chi-square results show that likelihood ratio statistics are highly significant (P < 00001), showing a strong explanatory power of the model. Among twelve explanatory variables hypothesized to be determine the local farmers’ attitudes towards wetlands conversion to farmland, seven of them were found to be significant. The ordered probit model output presented in Table 7 shows that age, total number of Tropical Livestock Unit (TLU), landholding size, and engagement in crop production had statistically significant influence on the local farmers’ attitudes towards wetland conversion at less than 1% probability level. On the other hand, participation in alternative livelihoods and mean farmers perception of wetland ecosystem services were significant at less than 5% probability level; while family size was significant at less than a 10% probability level.

**Table 7:** Ordered Probit Model parameter estimates of the factors influencing local farmers’ attitudes towards conversion of wetland to farmland.

Variables	Coef.	Std. Err.	P value
Age of the respondents	−0.0488	0.0064	0.000***
Gender of household head	−0.0462	0.3256	0.887
Educational Status	−0.0068	0.0431	0.874
Family size	0.0498	0.0333	0.150
Marital Status	−0.0248	0.1275	0.846
Engagement in crop production	0.9209	0.3225	0.004***
Engagement in off-or non-farm livelihood	−0.2863	0.1722	0.056*
Owning of farmland	−0.3835	0.2330	0.100
The landholding size	−0.7699	0.0974	0.000***
Livestock ownership	−0.2550	0.6042	0.673
Total number of livestock measured in TLU	−0.0674	0.0197	0.001***
Mean perception on the ecosystem service	−0.7486	0.3362	0.126

Source: Computed from household survey data. Note: * = significant at p < 0.1; ** = significant at p < 0.05; *** = significant at p < 0.001

In line with our prior expectations, farmers’ attitudes towards the wetlands’ change had an inverse relationship with the age of farmers, landholding size, and engagement in off/non-farm activities, livestock holding and perception on wetland ecosystem services, whereas it had a negative relationship with farmers’ dependency on crop production (see Table 7).

The attitudes of experts and department heads of the sample offices had also been significantly influenced by their educational status and the offices they work in (agriculture, land use and administration, environmental protection, and irrigation development offices). Working in agricultural, land administration, and irrigation offices had a statistically significant influence on experts’ attitudes towards wetland conversion at less than 1% probability level; whereas working in environmental protection offices and graduation in plant science had a statistically influence experts’ attitudes towards wetland change at less than 5% Table 8.

**Table 8:** Order prohibit parameter estimates of the factors influencing experts’ attitudes towards wetland conversion.

Variables	Coef.	Std. Err.	P value
Sex of the experts	−0.3451	0.4574	0.451
Age of experts	0.0270	0.0441	0.540
Educational status	−0.3497	0.2220	0.051*
Years of experience	−0.1802	0.2548	0.479
Working in an agricultural office	7.4089	1.1868	0.000***
Working in the livestock development office	6.2947	216.81	0.977
Working in the land administration & use office	4.6477	0.9496	0.000***
Working in the environmental protection office	−1.5091	0.6757	0.026**
Working in the irrigation development office	5.9061	1.0843	0.000***
Average perception on ecosystem services	−0.0125	0.3178	0.969

Source: Computed from expert survey data. Note: * = significant at p < 0.1; ** = significant at p < 0.05; *** = significant at p < 0.001

Working in the offices of agriculture, land use and administration, and irrigation development had positively influenced experts’ attitudes towards wetland cultivation. On the contrary, working in the environmental protection office had an inverse relationship with the attitudes of experts towards wetland change.

## 5. Discussions

The area of the three wetlands had decreased by 48% within 36 years. The extent of their loss was severe similar to the changes observed in other Ethiopian wetlands [4,13,18,54,55]. It is noted from LULC analysis that almost all parts of the lost wetlands were converted to farmland (2495 ha); the LULC transition matrix of that distinguishes these wetlands from the LULC trajectories of other wetlands found in Ethiopia. The findings of previous studies [4,13,18,54,55] in the other areas of the country showed that in addition to being transformed into farmland, wetlands have been converted to grasslands, built-up areas, and eucalyptus-dominated woodlots.

Studies [22] stated that the misconception of wetlands as “ wastelands” drives their conversion to farmland and other land use/covers. Our findings, however, indicated that wetlands were not considered as wastelands. Similar to their counterparts in other areas [56–58], the vast majority of households perceived the considerable role of wetlands in delivering provisioning services such as livestock watering, grazing pasture, seedling raising, thatching grass and reeds, food production, etc. Significant number of farmers has also acknowledged their role in providing some of regulating, supporting and cultural services. Similarly, experts/ department heads acknowledged the vital contribution of wetlands to the delivery of provisioning, supporting, regulating, and cultural services, which are in line with the prior research findings [57,59].

Despite acknowledging the ecosystem services provided by the study wetlands, 67.5% of households showed no interest in their conservation which is contradictory to the findings of the previous studies [56,60,61]. As reported by these studies, recognizing the direct and indirect benefits of wetlands fosters the interests of the local community to conserve these areas even to extent of making financial contribution.

The local households disinterest in our study wetlands was not also stemmed from the negative effects of wetlands. The qualitative data indicated that these wetlands did not harbor poisonous insects, primates, hyenas, or foxes. Consequently, the local community did not face threats from dangerous wildlife; their field crops were not threatened by crop-raiding primates; their livestock were not preyed by hyena or fox; and malaria rate showed no significant difference the adjacent and far villages. Hartter [25] and Mandishona & Knight [26] contend that the local communities are likely to develop negative attitudes towards wetlands and become less motivated to manage them when the areas serve as a habitat for wildlife that damage crops and threatened human and livestock; and when adjacent settlements experience a higher prevalence rate of malaria compared to the areas found far distance.

The disinterest of households in wetland management is closely associated with their inclination to reclaim these areas aimed at alleviating farmland shortages. The negative and statistically significant relationship between the landholding size and farmers attitude implies that households with minimal or no farmland were more likely to inclined toward wetland cultivation, a result that mirrors the findings from comparable some studies [11]. The inverse and significant relationship between age and households’ attitude towards wetland cultivation also suggests that landlessness and small landholding size increase a desire for wetland cultivation. As per survey findings, the vast majority of small landholders and landless individuals were younger households. Consistent with the previous findings [62] and our expectation, farmers with small herding size or no livestock assets were also more likely to farm wetland areas than farmers with larger herd size. The principal reason for higher inclination of farmers with small or no livestock could be that their less reliance on these ecosystems for livestock feed. Singh and Mistri [63] noted that the inclination toward wetland cultivation is higher among households with less reliance on wetland grazing than those whose livestock heavily rely on wetland grazing. Besides, the likelihood of wetland cultivation desire increase as the farmers’ dependency on crop farming grows. The inverse and significant association of off-farm and non-farm activities and households’ attitudes toward wetland cultivation suggests that depending only on crop farming could able to increase the likelihood of transforming wetlands to farmlands may be due to lack of alternative livelihoods. The corresponding observations were made by some authors [11,64] which reported that the farmers whose livelihoods depend on crop production were more engaged in the wetland cultivation than with farmers high livelihood diversification.

Contradictory with the findings of the previous research works [65], two-third of experts/department heads have also supported the conversion of the wetlands into farmland to mitigate rural unemployment. This indicates that the *woreda* administrations have considered wetland allocation to generate employment opportunities. The intention of allocating the wetlands to unemployed and landless households originates from the offices of agriculture, land use and land administration, and irrigation development. The statistically significant and positive association of experts’ attitude towards wetland cultivation with working in agricultural, land use and administration, and irrigation development offices is an important indicator for greater interests of these offices to the conversion of wetlands into farmland. On the contrary, the significant inverse relationship between experts’ attitude and working in the environmental protection office implies the demand of the environmental protection office for the preservation of wetlands over their conversion to agricultural land. This indicates the existence of a conflict of interest among different sector offices. Previous studies [37,66] also observed the existence of competing interests among government offices regarding the utilization of wetland ecosystems. These studies highlight that the conflicting interests of various government institutions create a significant challenge for effective wetland management and conservation.

The local governments’ interests to transform wetlands into croplands have materialized into the reality. The findings of the qualitative data revealed that the local government administrations have distributed wetlands for croplands to newly formed households. The distribution of wetlands for croplands was not the recent practice. As reported by informants, wetlands were allocated to newly formed households in the 1980s to avoid the frequent redistribution of the existing farmland. The allocation of wetlands, however, has been widely undertaken since the 1997 land redistribution. The shortage of farmland in the study *woredas* could not compel local authorities to allocate wetland areas to landless families and unemployed graduates. The data obtained from the two *woredas* land use and administration offices revealed that the average per household landholding size of Bure and Womberma *Woredas* in 2023 was 1.72 and 1.88 ha, respectively. This was more than twice Ethiopia’s national average and exceeded the Amhara region’s average by 0.73 to 0.89 hectares in 2022/2023. In terms of landholding size, over two-thirds of households possessed more than 1 hectare of land, which was higher than 37.1% and 23.2% of the corresponding figures of the national and regional levels [67], respectively. The factor that forced local government administrations to allocate wetlands was the stoppage of land redistribution in the Amhara Region. Amhara Regional State proclaimed the stoppage of land redistribution in any part of the region [68], irrespective of local land availability in specific *woredas*. Consequently, allocating wetlands for croplands was the only practical solution to the address the land request of landless young households and employ unemployed graduates. The practice of distributing communal to organized rural youths by local administration officials, aimed at resolving the shortage of farmlands, has also been observed in other areas of Ethiopia [37,69].

The distribution of communal lands including wetlands to landless households and unemployed youths was supported by policies and laws. The Ethiopian national employment policy and strategy [70] and the Amhara Region land administration proclamation (ANRS, 2006) provided the authority to the lower government administrations to allocate the communal lands to jobless youths. According to [70], the principal aim of communal land distribution including wetlands, is creating job opportunities in natural resource-based and non-farm activities. The data garnered through KIIS, however, showed that environmentally friendly livelihood activities were not practiced in the allocated wetlands. In contrast to our the findings, Negash et al. [71] and Meaza et al. [72] reported that the livelihood activities carried out in the allotted communal lands were eco-friendly. In our study areas, the parts of wetlands allotted for landless households and unemployed youths were cultivated to produce crops after their water receded similar to other wetlands [12,57,73,74]. Efforts have never been exerted to practice environmentally sound livelihood activities in the allotted wetland parts, corresponds with the empirical findings of Nabahungu & Visser [75] and Ondiek [64]. Similar to our study areas, Nabahungu & Visser [75] and Ondiek [64] observed that the local communities have largely neglected the application of eco-friendly or environmental sound livelihood activities. According to these studies, the governments of Rwanda and Kenya have promoted wetlands’ farming without due consideration of these ecosystems loss with the goal of mitigating food insecurity and improving the livelihoods of rural communities.

Along with the growing rate of landlessness, lack of job opportunities for TVET and university graduates heavily pressured local administrations to the allocate wetlands to unemployed youths. Reports from the micro-and small-scale enterprise office (WoMSSE) reveals that the *woreda* administrations have failed to create employment opportunities for the TVET and university graduates in small-scale business, cottage industry, agro-processing occupations, rural infrastructure maintenance services, and construction works mandated by the national employment strategy [70]. The difficulty of creating employment opportunities in off/non-farm sectors for job seekers have also been documented across various areas of Ethiopia [37,69].

The results of this study indicate that the transformation of the wetlands into cropland was not primarily driven by the need to fulfill the immediate needs. According to the survey, the average wheat and maize yields during the 2021/2 cropping season were 3.63 and 5.26 tones, respectively. This figure exceeded the corresponding national averages by 19.8% for wheat and 19.32% for maize. The yields of the two major crops grown in the study *woredas* were also higher by 28.3% and 23.1% than the regional (Amhara) average. The higher potential of the two woredas to produce wheat and maize was affirmed by Warner et al. [46]. As per the reports of Warner et al. [46], the two *woredas* were among the top ten wheat and maize producer *woredas* of Ethiopia. Consequently, they are significant suppliers of red pepper, maize, and wheat for the national and regional markets [45,46]. The analysis revealed that respondent farmers sold 43.8% and more than half of their wheat and maize production to the market, which was by far higher than the proportion of marketed cereal crops at the national level in the same cropping seasons. Supplying such a proportion of crops to the local to national market clearly showed that meeting the basic needs was not a significant issue in the study areas. This implies that the motivational factors (struggling for subsistence level of consumption) described by Maslow’s Hierarchy of Needs theory do not adequately explain wetland farming practice in the study areas.

## 6. Conclusions

Conversion of wetlands into farmland has been played substantial role for the continued degradation of wetlands. This study investigates the reasons why the significant areas of three wetlands have been converted to farmland in Bure and Womberma *Woredas* over the past 36 years despite providing tangible and non-tangible benefits. The study indicated that the wetlands’ conversion to farmland was primarily driven by the intense pressure exerted on the local authorities by young households following the stoppage of the existed farmlands. Besides, lack of employment opportunities for college and university graduates in both public and private sectors has prompted the *woreda* and *kebele* administration to view wetlands’ allocation as a vital job-creation strategy, which is endorsed by national rural development strategy and the region’s land use and administration law. The findings did not substantiate the claims that the nearby community converts wetlands into croplands to satisfy their daily needs. Meeting the basic needs was not a significant issue in the study areas since they are the major producers and suppliers of wheat and maize in the country. Since the local government administrations allocate wetlands for farming, individuals’ intention of maximizing benefits without the rules of engagement cannot be held responsible for wetlands’ conversion. Furthermore, this study found no evidence to validate the argument that the conversion of wetlands into agricultural land stems from viewing them as wastelands. Farm households and experts acknowledged the considerable role of the wetlands’ tangible and intangible benefits. Thus, government and development partners should expand rural off-farm livelihoods and urban job opportunities for young landless households and unemployed graduates to minimize ecological pressure on wetlands. Besides, evaluating the ecological conditions of wetlands is essential before transforming them into farmland to realize environmentally sustainable farming.

## Supporting information

S1 File Household Survey Questionnaire for Wetland Evaluation. (PDF)

S1 Table Perception of the community towards wetland ecosystem services. (DOCX)

S2 Table Attitude of the community towards the wetland fate and management. (DOCX)

S3 Table Expert perceptions on ecosystem services. (DOCX)

S4 Table Descriptive Statistics of Household Perceptions. (DOCX)

S5 Table Descriptive Statistics of the Household Attitudes Towards Management. (DOCX)

S6 Table Descriptive Statistics of Experts’ Perceptions and Attitudes on wetland ecosystem services and management. (DOCX)

S1 Fig The perception of the community towards wetland ecosystem services. (TIF)
