# Supplementary material for: Wetland conversion to farmland in Bure and Womberma Woredas, Northwestern Ethiopia: Implications for sustainable land use
Source: PLoS One. 2026 Jul 2;21(7):e0352888. doi: 10.1371/journal.pone.0352888 (PMC13327261; doi:10.1371/journal.pone.0352888)
Supplement: S1 File — (PDF) [file pone.0352888.s001.pdf]

S1_File. Household Survey Questionnaire for wetland evaluation

My name is Workiye Worie Assefa and I am currently a Ph.D. student and staff member at Bahir Dar University. I am conducting a study on the various benefits that wetlands provide to the local community, as well as the challenges they are currently facing. The aim of this study is to identify the factors that have led to the loss and degradation of wetlands in selected wetland watersheds in Bure and Wonberma districts. The information collected through the questionnaires will be used as part of my Ph.D. thesis. I have randomly selected a list of households from your village, and we found your name through the records of development agents/kebele administration. I kindly request your cooperation in answering questions about your land management practices, agricultural production, livelihood activities, and other related topics. Your honest and accurate responses are crucial for the success of this study and will be greatly appreciated. Please note that your participation is strictly confidential and your name will not be disclosed in any public documents. The information you provide will be used solely for the purpose of this study.

Thank you for your cooperation.

I. Identification

1.	Name of *Woreda/District/*	
2.	Name of kebele administration	
3.	Name of wetland	
4.	Name of the head of household	
5.	Date of survey	
6.	Name of enumerator	

II. Demographic and Social Characteristics

Age of respondent (in years)	Sex of respondent:	Educational level	Number of family members	Marital status
	**1.** Male<br>**2.** Female	1. None<br>2. 1-4<br>3. 5-8<br>4. 9-10<br>5. 11-12<br>6. TVET<br>7. University	Male _______<br>Female_____<br>Total____	1. Married or living together<br>2. Single<br>3. Divorced/separated<br>4. Widowed/widow

III. Means of livelihoods

Means of your household livelihoods? (Use key 1)	The amount of income generated in the last 12 years	How long been engaged in this livelihood activity	The trend of your dependency in this livelihood activity Increased [1] decreased [2] No change [3] No idea [4]

Key 1: Means of livelihoods

1. Crops production	5. Wage labor	8. brewing & selling of local alcohol
2. Livestock rearing	6. Petty trading	9. Making of craft from wetland materials
3. Salaried employment	7. Handicraft	10. Harvesting and marketing of wetland materials
4. Social grants	8. Remittance	11. Carpentry and or masonry
5. Land renting	9. Renting houses	12. Others, if any

IV. Land holding and use

1. Does your household own land? Yes [1] No [2]
2. If your answer is "Yes', could you please indicate the location, area, uses, etc in the table below?

Location of plot<br>1. Homestead<br>2. Wetland<br>3. Dryland<br>4. Wetland periphery	Number of plots	Total area (in hectares)	What are the plots used for? (Key 2)	For how many years have you cultivated the plot?	Mode of acquisition (key 3)	Has your land use right registered by woreda land use & administration?<br>Yes 1<br>No 2

Key 2: Arable land [1] grazing land [2] plantation purpose [3] others (if any, specify)
Key 3: Land re-distribution [1] inheritance [2] family gift [3] squatting [4] renting [5]

V. Crop production

1. Did you grow crop in 2020/21 harvesting year? Yes [1] No [2]
2. If yes, give the details of major crops grown in last cropping year

Location of plot	Type of crop grown (use key 4)	Area planted (ha)	Total output (kg)	Quantity sold (kg)	What was the price (in kg)	
Homestead						
Dry farmland						
Wetland						

Key 4. Types of crops grown

1. *Teff*	4.finger millet	7. faba bean	10. grass pea	12. potato
2. wheat	5. barely	8. cow pea	11. lentils	13. oats
2. Maize	6. Pepper	9. chickpea	12. oil seeds	14. others

3. Have you grown crops using irrigation? Yes [1] No [2]
4. If you produce crops using irrigation agriculture, give the details of crops produced in 2020, types of irrigation and source irrigation water in the following table

Location of plot	Type of crop grown (use key 5)	Area planted (ha)	Total output (kg)	What was the price (in kg)	Source of irrigation (use key 6)	Types of irrigation system (use key 7)	For how many years have you been irrigated?
Homestead							
Dry farmland							
Wetland							

Key 5. Types of crops grown

1. Maize	3.onion	5. tomato	8. garlic	10. pepper
2. wheat	5. potato	6.cabbage	9. barely	11. others

Key 6: Rivers/stream [1] springs [2] wetland [3] ponds [4] shallow wells [5] others

Key 7: Canals constructed by farmers [1[modern canal [2] diverting by motor pump [3] lifting by traditional methods [4] others (specify if any)

5. Have you planted trees and/or perennial crops in your farm plots? Yes [1] No [2]
6. If yes, please give response in the following table

Location of plots<br>1.home steads<br>2. dryland<br>3. wetland<br>4. Wetland edges	Types of trees/perennial crops<br>1. Eucalyptus tree<br>2. Cordial<br>3. *Khat*<br>4. Fruits<br>5. Sugar cane<br>6. Others (if any)	Area planted (ha)	Have you ever been produced?<br>Yes 1<br>No 2	If you started, total output (kg)	What was the price (in kg)	When it was planted (use key 8)

Key 8: One year ago [1] three years ago [2] five years ago [3] ten years ago [4]

7. If you grow crops and or planted fruits and trees, please give your response in the following tables

Farming activities	How long have you been practice it in the wetland?	The amount of money generated in the last 12 months	The trend of income generated from this farming activity over the year<br>No change [1] increased [2] decreased [3]
Cereal crops production			
Production of vegetables			
Fruit tree plantation			
Plantation of sugar cane			
Tree plantation			
Chat plantation			
Pepper seedling			

VI. Livestock Ownership

6.1. Does your household own livestock? Yes [1] No [2]

6.2.If yes, please mention the types and number of livestock you own and the trends of ownership the following table

Types of livestock	Has your household owned?<br>1=Yes<br>2=No	Number of owned	Trend of the number in the past 10 years<br>1=increased<br>2=decreased<br>3=No change
1. Oxen			
2. Cow			
3. Bull			
4. Heifer			
5. Sheep			
6. Goats			
7. Horse			
8. Mule			
9. Donkey			
10. Poultry			
11. Beehive			

6.8. Source of livestock feeding

Types of animal feed<br>1. Pasture grass<br>2. Crop straw<br>3. Crop after math<br>4. Hey<br>5. Fodder trees and grasses<br>6. By-products	The source of feed (key 9)	The current availability<br>1=Adequate<br>2=Inadequate	Frequency of feeding<br>1=every day<br>2=Seasonal<br>3=Rarely	The trends over the years<br>1=Increased<br>2= Decreased<br>3= No change

Key 9: The source of feed: communal grazing land [1] private grazing land [2] homestead [3] dry farm land [4] wetland [5] purchasing [6] others (specify if any)

6.9. If a wetland found near to your village is the source of feed for your livestock, in what way the livestock have fed the feed sources of a wetland?
Free grazing [1] rotational grazing [2] cut and carry system [3] hay [4]

6.10. If you late your livestock graze in the wetland, indicate the periods when you let your livestock graze/browse?

	Sept	Oct	Nov	Dec	Jan	Feb	March	April	May	June	July	Aug
Cattle												
Donkeys												
Goats												
Sheep												

6.11. If you are not let your livestock graze in the wetland, what are the reasons?
[1] Free grazing is prevented by community bylaws
[2] The local administration prohibited free grazing
[3] The macrophytes and grass species grown in the wetland are unpalatable
[4] Macrophytes and grass species of the wetland are inaccessible to livestock due to its water depth throughout the year
[5] grazing in the wetland make the livestock vulnerable to parasites and diseases
[6] others (specify, if any

6.12. If your livestock feed grasses or macrophytes of a wetland by other mode of feeding (rotational grazing, cut and carry system and hay), give your response for the questions provided below

Mode of feeding	Estimated value of used/harvested grass	How long have you been practiced?	
Controlled grazing			
Cut and carry system			
Hay			

VII. Water use

7.1. Which source of water do you use for the following activities during dry season?

Water for	Source (use key 10)	Location Homestead 1 Dryland 2 Wetland 3	Distance from home (in minutes)	Have you noticed any change in water supply in the last 10 years? Yes [1] No [2]	If yes, water supply Increased [1] Decreased [2]
Drinking					
cooking					
Washing cloths					
Bathing					
Washing household utensils					
Building purpose					
Watering of livestock					
Watering garden					
Others					

Key 10: Communal tap [1] developed spring [2] undeveloped spring [3] shallow well [4] protected hand dug well [5] pond [6] river/stream [7] others

7.2. Which source of water do you use for the following activities during wet season?

Water for	Source (use key 11)	Location Homestead 1 Dryland 2 Wetland 3	Distance from home (in minutes)	Have you noticed any change in water supply in the last 10 years? Yes [1] No [2]	If yes, water supply Increased [1] Decreased [2]
Drinking					
cooking					
Washing cloths					
Bathing					
Washing household utensils					
Building purpose					
Watering of livestock					
Watering garden					
Others					

Key 11: Communal tap [1] developed spring [2] undeveloped spring [3] shallow well [4] protected hand dug well [5] pond [6] river/stream [7] others

7.3. If your source of water for various purposes is a wetland and the supply of water decreased, what are the reasons?

[1] the volume of wetland water is decreased because of sedimentation
[2] the water of large area of the wetland is dried
[3] the volume of wetland water is decreased due to climate change
[4] the users of the wetland are alarmingly increased and hence create a shortage of water
[5] the diversion of wetland water for irrigation purpose is caused for the shortage of water for other purposes
[6] others (specify, if any)

7.4.If the water supply of wetland water for various purposes increased, what are the reasons?

[1] the volume of wetland water has increased because of an increasing of the summer rainfall
[2] the volume of wetland water increased as a result of increasing of river bank overflow to the wetland
[3] an increasing of ground water discharge
[4] others (specify, if any)

VIII. Use of wetland materials

8.5. Did your household collect wetland materials during the past cropping season?

Yes [1] No [2]

8.6.If yes, would please give the response for the question provided in the following table

Type of material	Did your household collect it? Yes 1 No 2	For what purpose Consumption 1 Sale 2	If your answer is" for sale", how much money did you earn in the last 12 months	How do you perceive the rate of your dependency over years? Increased [1] No change [2] decreased [3] No idea [4]
Firewood				
Edible fruits				
Thatch grass				
Craft material				
Fish				
Cheffe				
Sand harvesting				
Clay soils				
Others (specify, if any)				

8.7. Have you noticed any changes in the availability of wetland resources in the last ten years?

Yes [1] No [2]

8.8.If yes, give your response for the questions provided in the following tables?

Types of materials	Change in area coverage of resources Increased [1] Decreased [2]	Change in the availability of resource Increased [1] Decreased [2]	If the resource decreased in terms of area and availability, what are the reasons? (Use key 12)

Firewood			
Edible plants			
Thatch grasses			
Craft material			
Fish			
cheffe			
Clay soils			
Others (specify if any)			

Key 12: reasons for the decrease of wetland material in terms of area coverage and availability

1. the declining of the volume of wetland water due to low amount of rainfall	5.Over use of wetland material
2. the conversion of some part of wetland to farmland	6.the filling of wetland by sediments
3. misuse of wetland material	7.drying of wetland due to diversion of water for irrigation purpose
4. The loss of materials by invasive species infestation	8.others

8.9.Apart from wetland materials, do you get other benefits due to your proximity to the wetland? Yes [1] No [2]

8.10. If yes, which benefits are you aware of? Please select and indicate in the spaces provided the corresponding magnitude of these uses as they apply to your local wetlands.

Benefits	Very important	Important	Neutral	Less important	Not at all Important
Local climate stabilization (cooling effect)					
Sedimentation and make fertile soil for farming					
Purifying water for drinking					
Habitat of fauna and flora					
Center for recreation					
Center for tourist attraction					
Religious sites					
Cultural ceremonies					
Flood control					
Others (specify if any)					

IX. Ownership and management of wetland

9.11. Do you think that the wetland found in your local area has owner? Yes [1] No [2]

9.12.If your answer is "Yes", who is the owner of the wetland?

[1] few farmers who are living around of the wetland [2] the local community living in the villages around the wetland [3] the people who are living in the kebele administration in which the wetland is located [4] the kebele administration [5] Churches [6) woreda administration [7] others, specify if any

9.13.Is there any institution/ that administrate the utilization of wetland for various purpose? Yes [1] No [2]

9.14.If your answer is yes, what is/are these institutions?

[1] the committee organized by the local community [2] kebele land administration and use committee [3] wetland and other water related administrative committee [4] guards employed by kebele administration council [5] watershed management and administration committee [6] Iddir [7] religious institution [8] Civil societies [9] NGOs [10] others, if any specify

9.15. Is there governing rules/regulation that institution used to administer the wetland resource utilization? Yes [1] No [2]

9.16.If your answer is yes, which rules/regulations?

[1] the bylaw established by the local community [2] the bylaw established by kebele administrative council [3[the directives articulated for the enforcement of wetland policy [4] water resource management proclamation [5] watershed management and administration proclamation [6] others, specify if any

9.17.Is there any practice that undertaken the sustainable management of a wetland in your local area? Yes [1] No [2]

9.18.If your answer for Q 9.7 is "Yes", which management practices have implemented?

[1] using the wetland for a limited purpose [2] using only the matured wetland materials [3] protection of woodlands/shrubs and forests around the wetland [4] closing the wetland during wet season [5] preventing the wetland from livestock intervention [6] revegetation of wetland materials [7] controlling overexploitation of wetland resources [water, fish, grasses, etc) [8] controlling firing in the wetland [9] undertaking soil and water conservation structures in upper watersheds [10] others, specify if any

X. The fate of the wetland

10.19.How do you perceive the future fate of the wetland found in your locality?

[1] it will be shrunk/decreased [3] it will totally lost [5] I have no idea
[2] it will remain the same [4] it will be increased [6] others, specify if any

10.20.If your answer is either "will be lost" or "will be shrunk", what will be the reason?

[1] excessive diversion of wetland water for irrigation purpose will contribute for loss of wetland [2] the wetland is changing into farmland [3] the depth of a wetland is declining because of drought [4] the number of users of the wetland is an increasing so that create a burden [5] unwise of the wetland resources [6] there is no controlling system in the utilization of the wetland resource
[7] Others (specify, if any)

10.21.If your answer is "it will be increased or remain the same" what will be the reason for the sustainability of the wetland?

[1] The volume of wetland water has been increasing and preventing human and animal intervention [2] The wetland is protected by the community and government [3] the use of the wetland resource is governed by the regulations [4] others, specify if any

XI. Attitude of farms towards the wetland

11.1. Please give your response for the following question items by ticking (√)

Items	Strongly agree	Agree	No idea	agree	Strongly disagree
1. There is a scarcity of lands to produce enough food, therefore, people should be allowed to farm in the wetland					
2. Distribution of a wetland for landless youth households by local administration is the right action					

3. It is not good to allow uncontrolled livestock grazing in the wetlands					
6. A wetland is creating a favorable ground for mosquito; therefore, it should be converted to other uses					
7. A wetland is a habitat of birds, monkeys and apes so that my crops is damaged by these animals every year					
8. My livestock are eaten by jackals and hyena which are sheltered in the wetland; therefore, a wetland should be exploited					
9. The people in our village are bite and killed by snake and other poisonous animals which are breeding in the wetland so that a wetland should be destroyed					
10. The conversion of a wetland to other uses will create the shortage of water supply for various purposes so that it should be protected					
11. There should be a restriction in the use of fuel wood from a wetland					
12. Extraction of wetland materials should be controlled					
13. Using a wetland water for irrigation purpose bring a negative impact on the wetland so that it should be controlled					
14. Anybody who is living in our kebele or neighboring kebeles can use the wetland resources without any restriction					
15. The shrublands and forests around a wetland should be protected to prevent wetland degradation					
16. Using wetland for agricultural purpose out weights their uses for other purposes; therefore, I prefer to use for agriculture					
