# Supplementary material for: Wetland conversion to farmland in Bure and Womberma Woredas, Northwestern Ethiopia: Implications for sustainable land use
Source: PLoS One. 2026 Jul 2;21(7):e0352888. doi: 10.1371/journal.pone.0352888 (PMC13327261; doi:10.1371/journal.pone.0352888)
Supplement: S1 Table — (DOCX) [file pone.0352888.s002.docx]

**S1 Table**. Perception of the community towards wetland ecosystem services

| Ecosystem Services | Indicators | Very useful | less useful | Not useful | Don't know |
| --- | --- | --- | --- | --- | --- |
| Provisioning services | Crop production | 55.9 | 44.1 | 0 | 0 |
|  | Water for drinking | 43.2 | 56.8 | 0 | 0 |
|  | Water for other domestic use | 44.1 | 55.9 | 0 | 0 |
|  | Water for irrigation | 88.8 | 0 | 11.2 | 0 |
|  | Water for livestock | 100 | 0 | 0 | 0 |
|  | shelter for seedlings and crop aftermath | 100 | 0 | 0 | 0 |
|  | Pepper seedling raising | 100 | 0 | 0 | 0 |
|  | Sand for construction | 22.5 | 9.2 | 68.3 | 0 |
|  | Livestock grazing | 100 | 0 | 0 | 0 |
|  | Harvesting hey | 65.7 | 34.3 | 0 | 0 |
|  | Harvesting reeds for the greening of the floor | 100 | 0. 0 | 0 | 0 |
|  | Thatching for tukuls | 100 | 0 | 0 | 0 |
|  | Craft materials | 0 | 0 | 85 | 15 |
|  | Fishing | 0 | 0 | 100 | 0 |
|  | Firewood | 2.5 | 23.9 | 73.6 | 0 |
|  | Medicinal plants | 0 | 6.3 | 16.5 | 77.2 |
|  | Micro- climate regulation | 32.9 | 7.8 | 0.3 | 29.1 |
|  | Improving soil fertility | 34.3 | 32.3 | 12.5 | 20.7 |
|  | Water purification | 17.3 | 11.8 | 8.9 | 25.9 |
|  | habitat of wild animals | 21.6 | 15 | 6.6 | 16.4 |
|  | Recreational services | 23.3 | 18.7 | 11.8 | 17.6 |
|  | Tourist attraction | 2 | 13.8 | 45.2 | 35.7 |
|  | Flood regulation | 11.5 | 30.8 | 19 | 23.6 |
|  | Religious Services | 37.8 | 7.8 | 2.6 | 13 |
| Supporting services | Sediment retention | 34.5 | 32.3 | 12.5 | 20.7 |
|  | habitat of wild animals | 21.6 | 15 | 47 | 16.4 |
|  | Nutrient cycling | 0 | 0 | 0 | 100 |
|  | Support of pollination | 0 | 0 | 0 | 100 |
| Regulating services | Flood regulation | 11.5 | 30.8 | 34.1 | 23.6 |
|  | Water purification | 17.3 | 11.8 | 26.9 | 44 |
|  | Micro- climate regulation | 52.9 | 17.7 | 0.3 | 29.1 |
|  | Water regulation (storage) | 45.6 | 17.1 | 13.1 | 24.2 |
| Cultural services | Spiritual services | 67.8 | 16.6 | 2.6 | 13 |
|  | Recreational services | 51.9 | 18.7 | 11.8 | 17.6 |
|  | Tourist attraction | 2 | 13.8 | 45.2 | 39 |
|  | Research and education services | 0 | 0 | 0 | 100 |
|  | Aesthetic value | 0 | 29.3 | 0 | 70.7 |
