# Supplementary material for: Wetland conversion to farmland in Bure and Womberma Woredas, Northwestern Ethiopia: Implications for sustainable land use
Source: PLoS One. 2026 Jul 2;21(7):e0352888. doi: 10.1371/journal.pone.0352888 (PMC13327261; doi:10.1371/journal.pone.0352888)
Supplement: S2 Table — (DOCX) [file pone.0352888.s003.docx]

**S2 Table**. Attitude of the community towards the wetland fate and management

| Items | Strongly agree | Agree | No opinion | Disagree | Strongly agree |
| --- | --- | --- | --- | --- | --- |
| 1. Each farmer should be allowed to cultivate parts of the wetland due to the scarcity of land | 22.2 | 35.2 | 1.7 | 34.6 | 6.3 |
| 2.  Distribution of wetland to landless farmers is the right action | 33.1 | 40.1 | 0.3 | 21.6 | 4.9 |
| 3.  Wetlands should be protected for future use | 0.3 | 31.4 | 0 | 59.9 | 8.4 |
| 4.   Wetlands should be destroyed because |  |  |  |  |  |
| 4.1.  They allow mosquitoes to breed | 0 | 11 | 6.3 | 56.2 | 26.5 |
| 4.2.  They are a habitat of birds and primates that damage crops | 0 | 0.9 | 17.9 | 56.5 | 24.8 |
| 4.3.  They shelter jackals and hyenas | 0 | 0.3 | 8.1 | 62.5 | 29.1 |
| 4.4.  They harbor snakes and other poisonous animals | 0.6 | 0.9 | 8.1 | 64 | 265 |
| 5.  Wetland resources utilization should be regulated | 0.3 | 19.3 | 0.3 | 62.8 | 17.3 |
| 6. I prefer wetlands to be used for agriculture over other purposes | 29.1 | 18.2 | 1.7 | 31.1 | 9.8 |
