# Supplementary material for: Wetland conversion to farmland in Bure and Womberma Woredas, Northwestern Ethiopia: Implications for sustainable land use
Source: PLoS One. 2026 Jul 2;21(7):e0352888. doi: 10.1371/journal.pone.0352888 (PMC13327261; doi:10.1371/journal.pone.0352888)
Supplement: S3 Table — (DOCX) [file pone.0352888.s004.docx]

**S3 Table**. Expert perceptions on ecosystem services

| Ecosystem services | **Indicators** | Very useful | Less useful | Not use at all | don't **know** |
| --- | --- | --- | --- | --- | --- |
| Supporting services | Wild animal habitat | 0 | 18.9 | 6.8 | 55 |
|  | Nutrient Cycling | 60.8 | 39.2 | 0 | 0 |
|  | Support of pollination | 70.3 | 23 | 0 | 6.7 |
|  | Sediment retention | 81.1 | 0 | 0 | 14 |
| Cultural services | Religious festive celebration | 74.3 | 17 | 8.7 | 0 |
|  | Recreation service | 68.8 | 23.5 | 0 | 6.7 |
|  | Tourist attraction | 0 | 0 | 0 | 100 |
|  | Aesthetic values | 73 | 16.2 | 0 | 10.8 |
|  | Education and research services | 25.7 | 48.6 | 0 | 25.7 |
| Regulating services | Flood control | 0 | 13.5 | 29.7 | 18.8 |
|  | Local climate regulation | 91.9 | 0 | 0 | 8.1 |
|  | Water purification | 28.4 | 14.9 | 8.1 | 16.2 |
|  | Water regulation | 89.2 | 0 | 0.0 (0) | 10.8 |
| Provisioning services | Crop production | 100 | 0 | 0 | 0 |
|  | Water for drinking | 35.1 | 27 | 0 | 13 |
|  | Livestock water | 100 | 0 | 0 | 0 |
|  | Irrigation | 62.1 | 14.9 | 8.1 | 14.9 |
|  | Water for other domestic purposes | 55.4 | 18.9 | 8.1 | 17.6 |
|  | grazing pasture | 100 | 0 | 0 | 0 |
|  | Thatching for tukuls | 83.8 | 0 .0 | 0 | 16.2 |
|  | Firewood supply | 0 | 32.4 | 41.9 | 25.7 |
|  | Harvesting for the greening of the floor (*Cheffe*) | 100 | 0 | 0 | 0 |
|  | Harvesting hey | 58.1 | 37.8 | 0 | 4.1 |
|  | Wild foods | 0 | 14.9 | 45.9 | 39.2 |
|  | Craft materials | 0 | 0 | 37.2 | 62.8 |
|  | Sand extraction | 2.7 | 12.2 | 54.1 | 31 |
|  | Fishing | 0 | 0 | 100 | 0 |
|  | Medicinal plant | 0 | 17.6 | 0 | 82.4 |
