# Supplementary material for: Wetland conversion to farmland in Bure and Womberma Woredas, Northwestern Ethiopia: Implications for sustainable land use
Source: PLoS One. 2026 Jul 2;21(7):e0352888. doi: 10.1371/journal.pone.0352888 (PMC13327261; doi:10.1371/journal.pone.0352888)
Supplement: S4 Table — (DOCX) [file pone.0352888.s005.docx]

**S4 Table**. Descriptive Statistics of Household Perceptions

|  | N | Mean | Std. Deviation |
| --- | --- | --- | --- |
| **Provision services** |  |  |  |
| The wetland used for crop production | 347 | 1.44 | 0.498 |
| Used for drinking water | 347 | 1.63 | 0.484 |
| Water for domestic purpose | 347 | 1.59 | 0.493 |
| Water for irrigation | 347 | 1.48 | 0.854 |
| Water for livestock | 347 | 1.00 | 0.000 |
| used for the shelter of seedlings and feed | 347 | 1.00 | 0.000 |
| Used for seedlings of red pepper | 347 | 1.00 | 0.000 |
| Used for sand construction | 347 | 2.57 | 0.762 |
| Used for livestock grazing | 347 | 1.00 | 0.000 |
| Harvesting of livestock grass | 347 | 1.34 | 0.474 |
| Harvesting of grass for greening the floor | 347 | 1.00 | 0.000 |
| Harvesting for thatching | 347 | 1.00 | 0.000 |
| Harvesting craft mater | 347 | 3.15 | 0.360 |
| Firewood collection | 347 | 2.71 | 0.509 |
| Collection of medicinal plant | 347 | 2.71 | 0.578 |
| Valid N (listwise) | 347 |  |  |
| Regulating & supporting services | | | |
| Local climate regulation | 345 | 2.13 | 0.974 |
| Improve the fertility of the soil | 345 | 3.14 | 1.202 |
| Purify the water quality | 345 | 2.59 | 1.170 |
| use for different wild animals' habitats | 345 | 2.45 | 1.178 |
| Use for recreational activities | 345 | 2.68 | 1.336 |
| Tourist attraction (the center of tourism) | 345 | 3.99 | 1.045 |
| Center of religious activities | 345 | 1.99 | 1.030 |
| Center of cultural festive celebration | 345 | 2.87 | 1.448 |
| Prevention of floods | 345 | 3.33 | 1.267 |
| Valid N (listwise) | 345 |  |  |
