# Supplementary material for: Wetland conversion to farmland in Bure and Womberma Woredas, Northwestern Ethiopia: Implications for sustainable land use
Source: PLoS One. 2026 Jul 2;21(7):e0352888. doi: 10.1371/journal.pone.0352888 (PMC13327261; doi:10.1371/journal.pone.0352888)
Supplement: S5 Table — (DOCX) [file pone.0352888.s006.docx]

**S5 Table**. Descriptive Statistics of the Household Attitudes Towards Management

| **Descriptive Statistics** | | | |
| --- | --- | --- | --- |
|  | N | Mean | Std. Deviation |
| There is a scarcity of land to produce enough food; therefore, people should be allowed to farm in the wetland | 347 | 3.28 | 1.319 |
| Distribution of a wetland for landless youth households by the local administration is the right action | 347 | 2.25 | 1.258 |
| Wetlands should be protected for future use | 347 | 3.45 | 1.031 |
| A wetland creates a favorable ground for mosquitoes; therefore, it should be converted to other uses | 347 | 3.98 | 0.877 |
| A wetland is a habitat for birds, monkeys, and apes, so my crops are damaged by these animals every year | 347 | 4.05 | 0.678 |
| My livestock are eaten by jackals and hyenas, which are sheltered in the wetland; therefore, a wetland should be exploited | 347 | 4.20 | 0.585 |
| The people in our village are bitten and killed by snakes and other poisonous animals that breed in the wetland, so the wetland should be destroyed | 347 | 4.15 | 0.641 |
| The utilization of wetland resources (water, macrophytes, grasses) should be regulated | 347 | 3.78 | 0.962 |
| Using wetland for agricultural purpose out weights their uses for other purposes; therefore, I prefer to use it for agriculture | 347 | 2.64 | 1.424 |
| Valid N (listwise) | 347 |  |  |
