# Supplementary material for: Wetland conversion to farmland in Bure and Womberma Woredas, Northwestern Ethiopia: Implications for sustainable land use
Source: PLoS One. 2026 Jul 2;21(7):e0352888. doi: 10.1371/journal.pone.0352888 (PMC13327261; doi:10.1371/journal.pone.0352888)
Supplement: S6 Table — (DOCX) [file pone.0352888.s007.docx]

**S6 Table**. Descriptive Statistics of Experts' Perceptions and Attitudes on wetland ecosystem services and management

| **Descriptive Statistics** | | | | | |
| --- | --- | --- | --- | --- | --- |
|  | N | | Mean | Std. Deviation | |
| Provision services |  | |  |  | |
| Drinking water | 74 | | 2.47 | 1.397 | |
| Livestock water | 74 | | 1.59 | 0.826 | |
| Irrigation | 74 | | 2.39 | 1.280 | |
| Swimming | 74 | | 3.93 | 1.051 | |
| Washing clothes | 74 | | 2.68 | 1.160 | |
| Grazing pasture | 74 | | 1.55 | 0.830 | |
| Thatching grass | 74 | | 1.81 | 1.069 | |
| Crop production | 74 | | 3.18 | 1.369 | |
| Firewood supply | 74 | | 3.92 | 1.120 | |
| Cheffe/greening floor | 74 | | 1.45 | 0.779 | |
| Wild foods | 74 | | 4.01 | 1.066 | |
| Herbal medicine | 74 | | 0.06 | 0.000 | |
| Valid N (listwise) | 74 | |  |  | |
| Regulatory, Supportive, and Cultural Services |  | |  |  | |
| Sand extraction | 74 | | 4.14 | 1.077 | |
| Wild animal habitat | 74 | | 2.35 | 1.243 | |
| Religious festive celebration | 74 | | 2.77 | 1.360 | |
| Cultural festive | 74 | | 2.85 | 1.421 | |
| Recreational services | 74 | | 2.99 | 1.390 | |
| Flood control | 74 | | 2.45 | 1.305 | |
| Local climate regulation | 74 | | 1.64 | 0.915 | |
| Improving soil fertility | 74 | | 2.54 | 1.326 | |
| Purifying water | 74 | | 2.45 | 1.284 | |
| An adequate water supply | 74 | | 1.72 | 1.079 | |
| Valid N (listwise) | 74 | |  |  | |
| **Attitude** | | | | | |
| Wetlands should be cultivated due to the shortage of farmland for adequate production | 74 | 3.25 | | | 1.253 |
| The allocation of wetlands for landless youths is a correct action | 74 | 3.77 | | | 1.165 |
| The government should take action to protect wetlands | 74 | 2.82 | | | 1.254 |
| Wetland water should be diverted for the promotion of irrigation | 74 | 3.27 | | | 1.208 |
| Wetland cultivation is the only option for addressing rural unemployment | 74 | 3.95 | | | 1.262 |
| Valid N (listwise) | 74 |  | | |  |
