# Supplementary figures and images for: Wetland conversion to farmland in Bure and Womberma Woredas, Northwestern Ethiopia: Implications for sustainable land use
Source: PLoS One. 2026 Jul 2;21(7):e0352888. doi: 10.1371/journal.pone.0352888 (PMC13327261; doi:10.1371/journal.pone.0352888)

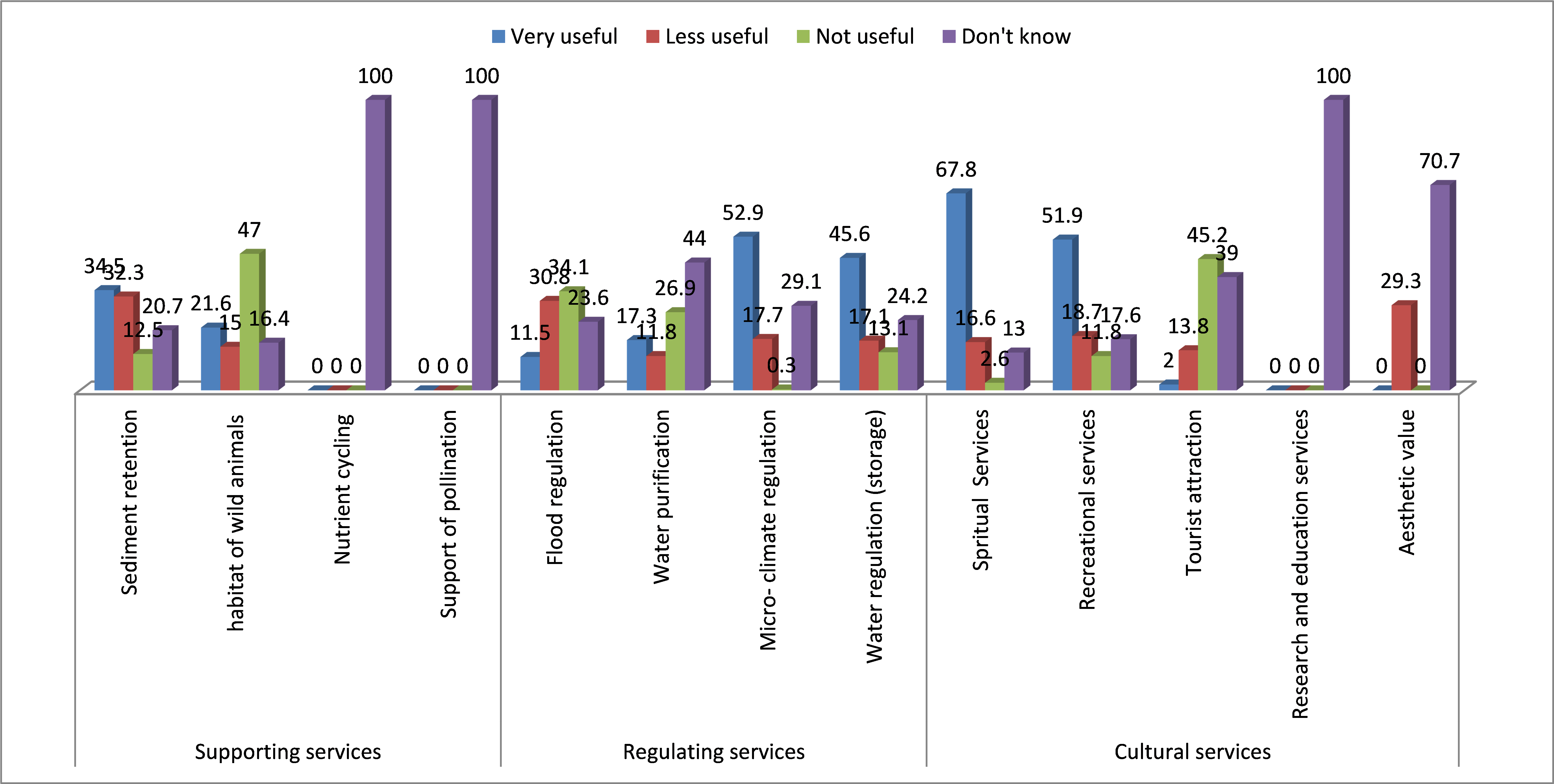

Supplement: S1 Fig — (TIF) [file pone.0352888.s008.tif]
