# Supplementary material for: Wetland conversion to farmland in Bure and Womberma Woredas, Northwestern Ethiopia: Implications for sustainable land use
Source: PLoS One. 2026 Jul 2;21(7):e0352888. doi: 10.1371/journal.pone.0352888 (PMC13327261; doi:10.1371/journal.pone.0352888)
Supplement: Attachment — Submitted filename: My comments nd suggestion.docx [file pone.0352888.s009.docx]

I would like to give my comment and suggestion for the manuscript

**Title:**

It needs to sightly modification to their `` Driving factors of wetlands into farmland for the sake of addressing landlessness and ……``

**Absract:**

The authors have presented a well-founded abstract; however, the structure of certain sentences could be refined to improve readability. Some sentences are overly complex or lack clear transitions between ideas, which may hinder comprehension. A careful review and restructuring of these sentences would strengthen the clarity of the abstract and provide readers with a more accessible context for the study.

**Background:**

The authors have presented a well-founded background however; the sequence of paragraph have been disordered. So, it needs to be reconstructed paragraph under background that will be focused: **The first paragraph** show that ecological and socioeconomical important of wetland in worldwide, Africa, Ethiopia and study sites.

**The second paragraph** will show to negative impact and community perception on the wetland

**The third paragraph** will consist of driving factors of wetland into foreland

**The fourth paragraph** will be written the effect of farmland than wetland resource or viceversal

**The fifth paragraph** will be showed to limitation of data, driving factors of wetland into farmland, current percentage of wetland and the action treatment of government and community participation

**Materials and Methods**

The authors have presented the study sites. However, it needs to seasonal variation, estimate of water volume, source of water, association linked with other dams, shedding areas GPS data, reference in 1985, 1995, 2010 and 2021. Additionally, authors will mention about estimation of human population size and grazing animals

**Methods of data collection**

**Authors will mention the shapefiles receded year**

**Survey Questionnaire**

It need clarify the sort out the list of question with in the categories

Wetland areas were not mentioned or data collection methods to their useful and harmful for the community

**Data analysis**

Authors will sort out dependent variable (Y) output and independent variables (x) factors clearly**.**

**Results**

Authors did not mention loss of plants and animals’ species and also other ecological damage when wetland converted to farmland. On other hands, these was not mentioned to avoid the community problems or remined the same or increasing to in the area

**Discussion**

Your discussion and conclusion were antagonistic each other in same parts but not all. So, it needs to clarification and mention based on your results
