# Supplementary material for: Wetland conversion to farmland in Bure and Womberma Woredas, Northwestern Ethiopia: Implications for sustainable land use
Source: PLoS One. 2026 Jul 2;21(7):e0352888. doi: 10.1371/journal.pone.0352888 (PMC13327261; doi:10.1371/journal.pone.0352888)
Supplement: Attachment — Submitted filename: A rebuttal letter.docx [file pone.0352888.s010.docx]

**Rebuttal letter**

**We** sincerely **thank an academic editor and reviewers** for your time and efforts to comment on our article and provide **constructive comments** to further improve its quality. We have made our effort to incorporate the responses of the comments provided by academic and reviewers. The responses are reported un a rebuttal letter

Response to academic editor

1. Please ensure that your manuscript meets PLOS ONE's style requirements, including those for file naming. The PLOS ONE style templates can be found at

**Response: we prepared the manuscript based on PLOSE ONE style**

2. Please provide additional details regarding participant consent. In the ethics statement in the Methods and online submission information, please ensure that you have specified (1) whether consent was informed and (2) what type you obtained (for instance, written or verbal, and if verbal, how it was documented and witnessed). If your study included minors, state whether you obtained consent from parents or guardians. If the need for consent was waived by the ethics committee, please include this information.

**Response:** thank you for your suggestion. We have done based on your recommendation. The ethical approval has incorporated revised manuscript under research methods section. The explanation is put as follows

**Ethical approval**

The ethical aspects of this research proposal were evaluated and approved by the review committees of the Blue Nile Water Institute and Research and Community Engagement Vice Office in accordance with Bahir Dar University's 2022 research guideline.  After the approval of the review committees, a letter of requesting support was written from the office of the University Research and Community Engagement Vice President and then, the permission letters were obtained from the study *woredas*.

After obtaining permission from the woreds’ sector offices, research participants were fully informed about the purpose of the research, and how the data obtained from them would be used. Informed consents were then requested from respondents (participants), key informants and FGD participants. Each person provided their unwritten, free assent to participate, having demonstrated an understanding of the study's implications and their right to refuse.

3. Please remove any funding-related text from the manuscript and let us know how you would like to update your Funding Statement. Currently, your Funding Statement reads as follows:

**Response: We have removed funding-related text from the manuscript**

4. Please state what role the funders took in the study.  If the funders had no role, please state

**Response:-** The funders had no role in study design, data collection and analysis, decision to publish, or preparation of the manuscript

5. Please confirm at this time whether or not your submission contains all raw data required to replicate the results of your study. Authors must share the “minimal data set” for their submission. PLOS defines the minimal data set to consist of the data required to replicate all study findings reported in the article, as well as related metadata and methods (<https://journals.plos.org/plosone/s/data-availability#loc-minimal-data-set-definition>).

**Response: we agree with your idea and hence the data are available subject to a formal request to the authors**

6. Please ensure that you refer to Figures 5, 6, 7  and 8 in your text as, if accepted, production will need this reference to link the reader to the figure.

**Response: sorry for the errors we made. Now we have made corrections**

7. We note that Figures 1, 2, 3, and 4 in your submission contain map and satellite images which may be copyrighted. All PLOS content is published under the Creative Commons Attribution License (CC BY 4.0), which means that the manuscript, images, and Supporting Information files will be freely available online, and any third party is permitted to access, download, copy, distribute, and use these materials in any way, even commercially, with proper attribution. For these reasons, we cannot publish previously copyrighted maps or satellite images created using proprietary data, such as Google software (Google Maps, Street View, and Earth). For more information, see our copyright guidelines: <http://journals.plos.org/plosone/s/licenses-and-copyright>.

Response. Figure 1 has been produced by us using shapefiles and a basemap. These data are freely available but require acknowledging their source. Therefore, we provided the source credit both in the figure and the figure caption as follows: Fig. 1. Location map of the study wetlands (Source attribution: Service Layer Credits: © OpenStreetMap (and) contributors, CC-BY-SA; Source: Esri, Maxar, Earthstar Geographics, and the GIS User Community).

Figures 2-4 have been produced using Landsat satellite images, which are freely available, and we acknowledge as follows:

Fig 2. Spatiotemporal cover change of the Foket floodplain wetland (data source: Landsat TM (1985 & 1995), Landsat 7 ETM+ (2000 & 2010), and Landsat-8 OLI (2021) images courtesy of the US Geological Survey)

Figure 3: Spatiotemporal cover change of the Kotlan wetland, specifically to its downstream portion (data source: Landsat TM (1985 & 1995), Landsat 7 ETM+ (2000 & 2010), and Landsat-8 OLI (2021) images courtesy of the US Geological Survey)

Figure 4: Spatiotemporal cover change of Wadera Wetland (data source: Landsat TM (1985 & 1995), Landsat 7 ETM+ (2000 & 2010), and Landsat-8 OLI (2021) images courtesy of the US Geological Survey)

8. Please provide enough detail on the Landsat methodology.

Response: We have provided detailed information on Landsat image analysis methodology in the revised manuscript (table is included)

**Table 1.** Characteristics of satellite images used to analyze LULC of wetlands

| Year acquired | Satellite | Sensor | Spatial resolution | Path/Row | Bands used & its range of wavelengths | |
| --- | --- | --- | --- | --- | --- | --- |
|  |  |  |  |  | Bands | Wave length |
| 1985, 1995, 2010 | Landsat 5 | MSS/TM | 30m × 30m | 170/53 | 1, 2, 3, 4, 5 & 7 | 0.45-2.35µm |
| 2000 | Landsat 7 | ETM+ | 30m × 30m | 170/53 | 1, 2, 3, 4, 5 & 7 | 0.45-2.35µm |
| 2021 | Landsat 8 | OLIS/TIRS | 30m × 30m | 170/53 | 2, 3, 4, 5, 6 & 7 | 0.45-2.25µm |

9. Which Landsat sensors were used (TM, ETM+, OLI)?

Response: It has already been stated in the manuscript lines number 197-199 as we used Landsat image series, including Landsat 5 Thematic Mapper (TM), Landsat 7 Enhanced Thematic Mapper Plus (ETM+), and Landsat 8 Operational Land Imager (OLI/TIRS).

10. What were the pre-processing steps?

Response: The pre-processing steps have already been elaborated in the manuscript from line 2019 to 2015 as” All datasets are Level 2, Collection 2, Tier 1, which have undergone geometric, radiometric, and atmospheric corrections by the image provider (U.S. Geological Survey) and are thus considered the highest quality. From these collections, we selected Bands 1–7 (excluding Band 6) from Landsat 5 and Landsat 7, and Bands 2–7 from Landsat 8. Only images with less than 10% cloud cover were composited in GEE for each year from January 1 to December 30, with the median value applied based on the geometry of the study region.”

11. Which classification method was used?

Response: Thank you; we included the following additional texts to accommodate the reviewer and editor concerns:

The classification procedure in this study involved the following steps:

1. Acquiring sufficient training samples for each land cover class (50-60 samples per class)
2. Creating signature files by combining the stacked images with the training samples
3. Using the signature files to classify the images into their respective land cover classes through maximum likelihood supervised classification

The identified land cover classes included water, dense vegetation, low vegetation, farmland covered by crop residue, and plowed farmland. The classification results were then reclassified into wetland and cultivated land using spatial analysis tools in ArcGIS 10.8.

During the digitization of training samples, careful consideration was given to selecting representative sites with sufficient pixel coverage, as the wetland areas were of a manageable size. High-resolution imagery from Google Earth Pro was consulted to ensure accuracy, and the classification results demonstrated excellent accuracy.

11. Accuracy assessment is missing.

Response: We did the accuracy assessment, but failed to include it in the manuscript. Therefore. We included it in the revised manuscript.

The overall accuracy and a Kappa analysis (equation 1) were performed to validate the each land cover class of the study periods (1985, 1995, 2000, 2010 and 2021) based on the confusion matrix. The confusion matrix shows the user’s accuracy, the producer’s accuracy, and the Kappa coefficient. Users’ accuracy is the probability that the land cover classes identified on the map is classified into the same category on the ground, whereas producer’s accuracy is the probability that the land covers identified on the ground is classified into the same category on the map (Lillesand et al., 2015). The confusion matrix compared each pixel in the reference map with its corresponding pixel in the classified map.

$$Kappa=\frac{observedaccuracy-chanceofagreement}{1-chanceofagreement}$$

The confusion matrix reveals that an overall accuracy of LULC for four reference years (1985, 1995, 2000 and 2010) was 95.50 % for 2021 was 97.27%. The Kappa coefficient of the LULC for the five reference years were 78.15, 79.93, 81.72, 88.96 and 87.19%, respectively. These indicate strong agreement between the classification and reference data. High classification accuracy was observed across both LULCs, with user's accuracy of the reference years ranging from 86.67% to 96.66% and producer's accuracy spanning from 86.79% to 94.92%, demonstrating strong reliability in identifying wetlands and cultivated land.

Responses for reviewers

Reviewer #1:

1. The abstract parts mostly focused on Excessive Focus on Negation and Insufficient Elaboration of the Key Finding Hence improved these parts from abstract.

**Response:-thank you very much for your valuable comment. We have improved the abstract**.

2. The study involved Field research (collecting data in a natural setting). While the focus is social science make Clarify if any specific regional/local administrative permission was obtained for field research and KIIs in the Woredas (Bure and Womberma).

**Response: the permission was obtained from the two woreda sector offices based on the request of the university. It is included in the ethical approval part of the modified manuscript**

Reviewer #2

1. The title needs to slightly modification to their `` Driving factors of wetlands into farmland for the sake of addressing landlessness and ….”

**Response**:- We appreciate your valuable perspective. We have reached an agreement on the modification of the title itself, but the proposed title did not convince. Thus we proposed our title “*Transforming wetland ecosystem into farmland in Bure and Womberma Woredas, Northwestern Ethiopia: a strategy for job creation and alleviating land shortages”*

2. The authors have presented a well-founded abstract; however, the structure of certain sentences could be refined to improve readability. Some sentences are overly complex or lack clear transitions between ideas, which may hinder comprehension. A careful review and restructuring of these sentences would strengthen the clarity of the abstract and provide readers with a more accessible context for the study.

**Response: thank you for fruitful comments. We have made a modification based on your request**

3. The authors have presented a well-founded background however; the sequence of paragraph have been disordered. So, it needs to be reconstructed paragraph under background that will be focused: **The first paragraph** show that ecological and socioeconomical important of wetland in worldwide, Africa, Ethiopia and study sites

**Response: Thank you for that clarity. We have done based on your recommendation. The wetland coverage from global to Ethiopian context and their ecological and socioeconomical importances are reported in the first paragraph ( see below)**

Wetlands are the vital landscapes of the earth’s surface. They covered an area of over **12 to 16 million km²**, which represents approximately **6% to 8%** of the planet's total land area [1]. **African wetlands comprise about a tenth of the planet's total wetland area, making the continent a major contributor to these crucial ecosystems** [2,3]. Ethiopia is recognized as one of African countries which endowed with significant and diverse wetland resources. Assefa and Eneyew [4] estimated the extent of the wetlands of the country at around 26,424 km² or 2% of the country's total land area. These wetlands provide diverse benefits, from tangible resources like water, food, and building materials to intangible values such as flood control, climate regulation, and cultural significance. The ecosystem services delivered by wetlands vary across different spatial scales, from global to local [5]. At global level, they play an important role in the provision of important ecosystem services like the provision of clean water, adaptation to climate change, and support for biodiversity [6]. At the local level, wetlands play a crucial role in sustaining the livelihoods of community and ensuring food security by providing materials in addition to their crucial roles in regulation and support services. The contribution of wetlands in sustaining the community's livelihoods and household food security is more important in Africa including Ethiopia. Millions of people in Africa, including Ethiopia, heavily depend on the essential **provisioning services** delivered by wetlands for their survival and livelihoods [1].

**The second paragraph** will show to negative impact and community perception on the wetland

**Response;- Thank you very much for your insightful feedback. We have done improvement based on your suggestion. The important issues discussed in the paragraph two the loss of wetlands from global to local level (see below)**

Despite the essential services they provide, wetlands are declining at alarming rates faster than any other ecosystems [7]. According to Darrah et al. [8], the global natural wetlands’ area shrank by roughly 35% from 1970 to 2015, with the rate of decline accelerating after the year 2000. The disappearance of inland wetlands was higher in developing countries particularly in Asia and Africa [1]. Like many other African nations, **Ethiopia has experienced significant loss and degradation of its wetlands** over the last two decades. Assefa and Eneyew [4] reported a 20% reduction in freshwater wetland coverage of the country from 2000 to 2020.

**The third paragraph** will consist of driving factors of wetland into foreland

Response:- we have also made some modification. This paragraph focuses on the driving factors of wetland loss or degradation in general including agriculture. We showed agriculture significant contribution to wetland degradation (see below)

Numerous factors are the principal drivers behind the accelerated **loss and deterioration of global wetland ecosystems**. Agriculture, urbanization, aquaculture and industry are reported to be major drivers of global wetlands’ loss. Ballut-Dajud et al. [9] indicated that agriculture took the lion share (25%) for the loss **and deterioration of global wetland ecosystems. The contribution of agricultural encroachment and intensive cultivation to inland wetland loss in African countries, including Ethiopia, is substantial** [9,10]. Assefa and Eneyew [4] indicated that farmland expansion had taken a lion share for the loss or degradation of wetlands in the country. The studies done at the micro-level have also reported the contribution of farmland expansion to the loss of 20 to 90 % of wetland areas in the past three decades [4,11–14].

Paragraph five

**Response: No change was made on the paragraph five. It is aligned with your recommendation**

**Materials and Methods**

4. The authors have presented the study sites. However, it needs to seasonal variation, estimate of water volume, source of water, association linked with other dams, shedding areas GPS data, reference in 1985, 1995, 2010 and 2021.

Response: We don’t have data on the volume of water of the wetlands. But, incorporate the seasonal variation and the source of water as follows

The wetlands under study are located within river channels, making them **riverine.** Surface water during rainy season and groundwater are the main water sources for these wetlands. Their water levels are typically high between July and December, and many of their parts recede from January to June.

5. Additionally, authors will mention about estimation of human population size and grazing animals

**Response:- the total population of the two *woredas* is mention as follows based on your suggestion**.

Central Statistical Service [48] projected that the total population of Bure and Womberma Woreda in 2021 was 171,792 and 126,242, respectively. Of these, 68,209 people were the residents of the *kebele* administrations located adjacent to the wetlands.

**The grazing animals estimation is also mentioned as**

Based on the agricultural offices of the two *woredas*, 67095.3 TLU were kept in the *kebele* administrations which are adjacent to the wetlands [11]

6. **Authors will mention the shapefiles receded year**

**Response: we have done based on the request**

7. It need clarify the sort out the list of question with in the categories

**Response: We mention as requested**

The question items incorporated under household profile section consists of age, sex, marital status, education background, family size and livelihood activities. Age, marital status, sex and educational background were also included under personal profile section of expert’s questionnaire in addition to their working experience, position held, and field of specialization. The question items in the wetland ecosystem perception sections for both households and experts’ questionnaires covered the importance of wetlands in the delivery of provisioning, regulating, supportive and cultural services. The attitude section of the questionnaires for both experts and households incorporated the items concerning opinions on wetland cultivation and sustainable management.  Likert-scale type questions were **prepared** to **elicit** households’ and experts'/department heads' perceptions of wetland ecosystems and their attitudes toward wetland cultivation.

8. Data analysis: authors will sort out dependent variable (Y) output and independent variables (x) factors clearly

Response; we have tried to sort out the ependent variable (Y) output and independent variables (x) factors clearly as follows

Suppose the households and experts’ attitude i ($Y_{i}$ ) is the unobserved variable (latent variable) and is expressed in the following equations ($Q_{1}$ and$Q_{2}$)

$HHAT$=$\beta_{0}$+$\beta_{1}*HHG$+$\beta_{2}*MHH$+$\beta_{3}$ * HHAG+$\beta_{4}*FSIZ$+$\beta_{5}*EDUCH$+$\beta_{6}$*CRENG+$\beta_{7}*OFFEN$+$\beta_{8}*FAROWN$+$\beta_{9}*LASIZ$+$\beta_{10}*LIVEOWN$+$\beta_{11}*LIVESIZ$+$\beta_{12}*MEANPERC$+£…………………………$Q_{1}$

Where $HHAT$= household respondent attitude towards wetland cultivation, $\beta_{0}$ = is a constant, $\beta_{i}$ is representing the coefficient of independent variables described in Table 2, and ε is the error term.

$HHAT$=$\beta_{0}$+$\beta_{1}*EXSEX$+$\beta_{2}*AGEXP$+$\beta_{3}$ * +$\beta_{4}*FSIZ$+$\beta_{5}*EDUEXP$+$\beta_{6}$*EXPY+$\beta_{7}*AGRIO$+$\beta_{8}*LFDO$+$\beta_{9}*LUAO$+$\beta_{10}*ENVPRO$+$\beta_{11}*MEANPERC$+£…………………………………………………$Q_{2}$

Where, $EXAT$= experts’ attitudes; β_0_ is a constant, β_i_ is the coefficient of independent variables described in Table 3, and ε is the error term.

9. **Results:-**Authors did not mention loss of plants and animals’ species and also other ecological damage when wetland converted to farmland. On other hands, these were not mentioned to avoid the community problems or remained the same or increasing to in the area

**Response:- the assessing or analyzing loss of plants and animals’ species as well as ecological damage was one our study objectives. So, we did not incorporate in the results. But, we included the implication of wetlands’ cultivation on loss of plants and animals’ species and ecological degradation like the following**

The transformation of wetlands into farmland has profound negative implications for flora and fauna, primarily through **habitat loss.** During interviews, elder informants witnessed the threatening of wild animals because of the conversation of wetlands into farmlands. According to them, the macrophytes and woody plants of the wetlands were dense prior to three decades ago. They harbored antelopes, monkeys, various bird species, etc. The findings of KII reported that the density of macrophytes and woody plants, however, have been decreased and thereby the loss of mammals, reptiles and birds. The wetlands’ cultivation has also negative implication on the hydrological functioning of wetlands**.** During a field survey, it was noted that the water from the wetlands was redirected to farmlands for irrigation. The diversion of water from wetlands has a major impact on reducing water volume, particularly during dry seasons.

10. **Discussion:-**Your discussion and conclusion were antagonistic each other in same parts but not all. So, it needs to clarification and mention based on your results

**Response:**-Thank you for your insightful observation. We found the some contradiction between discussion and conclusion. The contradictions were (1) the severity of farmland expansion. In the discussion part, the magnitude of cultivation reported as it is not sever but the reverse is true in the conclusion. The other contraction was observed in relation to the factors wetland cultivation. We have tried to avoid the contradition
