# Supplementary material for: Wetland conversion to farmland in Bure and Womberma Woredas, Northwestern Ethiopia: Implications for sustainable land use
Source: PLoS One. 2026 Jul 2;21(7):e0352888. doi: 10.1371/journal.pone.0352888 (PMC13327261; doi:10.1371/journal.pone.0352888)
Supplement: Attachment — Submitted filename: Comment and suggestion to this review paper.docx [file pone.0352888.s011.docx]

Comment and suggestion to this review paper `` Transforming wetland ecosystem into farmland in Bure and Womberma Woredas, Northwestern Ethiopia: a strategy for job creation and alleviating land shortages``.

**Title:**

Here are several improved and more appealing title options that avoid the phrase “job creation and alleviating land shortages” while keeping the meaning strong and professional: Wetland Conversion to Farmland in Bure and Womberma Woredas, Northwestern Ethiopia: Implications for Sustainable Land Use. If you want it to sound more academic, more policy-focused.

**Abstract:**

Wetland cultivation is influenced by the characteristics of both households and experts. Due to limited farmland availability, farmers often expand cultivation into wetlands without fully considering agroecological principles. Additionally, insights from multidisciplinary experts provide important perspectives on this issue. Therefore, it is essential to incorporate agroecologists’ evaluations when assessing the conversion of wetlands into farmland, ensuring that decisions are made in line with environmental conditions.

**Introduction:**

The authors did not address the beneficial and adverse effects of wetlands on agroecology in the study sites. Moreover, there is a strong likelihood that wetlands may be converted into farmland due to prevailing environmental conditions. Additionally, the authors failed to clearly state the study objectives in the final paragraph of the introduction section.

**Research Methods:**

The authors adequately describe the research methodology. However, they fail to specify data analysis techniques or the appropriate software used to clearly distinguish dependent variables respect to independent variables analyses. In addition, that rewrite the framework of independent variables under methodology part but no writes result and data analyzed

**Results:**

Authors should be rewriting only the results but not describe of methodology

**Discussion and Conclusion:** It need to rewriting and to make smoothly
