# Supplementary material for: Wetland conversion to farmland in Bure and Womberma Woredas, Northwestern Ethiopia: Implications for sustainable land use
Source: PLoS One. 2026 Jul 2;21(7):e0352888. doi: 10.1371/journal.pone.0352888 (PMC13327261; doi:10.1371/journal.pone.0352888)
Supplement: Attachment — Submitted filename: Reviewer Comment and suggestion to this paper.docx [file pone.0352888.s012.docx]

**Reviewer Comment and suggestion to this paper**

**Title:** “Transforming Wetland Ecosystems into Farmland in Bure and Womberma Woredas, Northwestern Ethiopia”

Several improved and more compelling title options can be considered to enhance clarity and academic strength while avoiding the phrase “job creation and alleviating land shortages.“Wetland Conversion to Farmland in Bure and Womberma Woredas, Northwestern Ethiopia: This version provides a more scholarly and policy-oriented tone.

**Abstract:**

Wetland cultivation is shaped by both household characteristics and expert perspectives. Due to limited availability of farmland, farmers often expand agricultural activities into wetlands without adequately considering agroecological principles. Furthermore, insights from multidisciplinary experts offer valuable perspectives on this issue. Therefore, it is crucial to integrate agroecological evaluations when assessing wetland conversion to farmland, ensuring that decisions align with environmental sustainability.

**Introduction:**
The introduction does not sufficiently address both the positive and negative impacts of wetlands on agroecological systems within the study areas. There is also a strong possibility that wetlands are being converted into farmland due to existing environmental and socio-economic pressures. Additionally, the study lacks clearly stated objectives in the concluding paragraph of the introduction, which weakens the overall focus of the paper.

**Research Methods:**

While the research methodology is generally well described, the authors do not clearly specify the data analysis techniques or the software used. There is also insufficient explanation of how dependent and independent variables were analyzed. Although a framework for independent variables is presented, the paper does not clearly link this framework to the results or data analysis.

**Results:**
The results section needs substantial revision. Instead of presenting and interpreting findings, the authors continue describing methodological aspects. This section should focus strictly on the study findings, supported by appropriate data presentation.

**Discussion and Conclusion:**

Both sections require significant rewriting to improve clarity, coherence, and depth of analysis. The discussion should critically interpret the findings in relation to existing literature, while the conclusion should clearly summarize key insights and provide meaningful recommendations.
