# Supplementary material for: Wetland conversion to farmland in Bure and Womberma Woredas, Northwestern Ethiopia: Implications for sustainable land use
Source: PLoS One. 2026 Jul 2;21(7):e0352888. doi: 10.1371/journal.pone.0352888 (PMC13327261; doi:10.1371/journal.pone.0352888)
Supplement: Attachment — Submitted filename: Rebuttal letter second revision.docx [file pone.0352888.s013.docx]

**Rebuttal letter second revision**

**We** sincerely **appreciate the academic editor and reviewers** for dedicating their time and providing valuable **feedback** to help us improve our manuscript. We have revised the manuscript to fully address the feedback and suggestions provided by academic editor and reviewers. The responses for the feedback are reported as follows

**Responses to academic editor comments**

1. In the theoretical section, the inclusion of many theories makes it feel scattered; I suggest focusing on one or two key frameworks and linking them clearly for consistency.

**Response:** Your observation is critical. We also understood the lack of focus of the theoretical framework part before your feedback. We avoid three theoretical arguments and we focus on three theoretical issue. Our analysis has based on these three theories i.e. institutional theory, tragedy of the commons, and Maslow's Hierarchy of Needs

2. revise for simple, direct language and remove redundancy, especially in the introduction and theoretical sections, as the manuscript still needs improvement in writing clarity, grammar, and flow; many sentences are long and repetitive, making it hard to follow.

**Response**: we have made our efforts to avoid redundancy, to shorten the long sentences and correct the grammatical errors. We believe that much improvement has been done to make the manuscript easily readable

3. Improve organization by tightening the background and theoretical section.

Response: Done based on suggestion

4. The discussion should better link findings with existing literature.

Response: Done based on your suggestion

5. The conclusion is somewhat long (repetitive of the results), make it short and concise.

**Response: We have made effort to make the conclusion concise and to avoid repetion**

6. There are also some irrelevant references; reduce and refine the reference list.

**Response: The irrelevant references are avoided or reduced**

**The responses for reviewers**

**Reviewer 1**

1. The title of the manuscript should be modified

Response: the title is modified based on your suggestion “Wetland Conversion to Farmland in Bure and Womberma *Woredas*, Northwestern Ethiopia: Implications for Sustainable Land Use”

2. The abstract should be written as:

“Wetland cultivation is shaped by both household characteristics and expert perspectives. Due to limited availability of farmland, farmers often expand agricultural activities into wetlands without adequately considering agroecological principles. Furthermore, insights from multidisciplinary experts offer valuable perspectives on this issue. Therefore, it is crucial to integrate agroecological evaluations when assessing wetland conversion to farmland, ensuring that decisions align with environmental sustainability.”

**Response**: your observation on the limitations of the abstract is absolutely acceptable. However, the suggested abstract does not clearly provide overall picture of the study. We have totally modified the abstract part as:

*This study investigates the reasons why wetlands transformed to farmland. The data were garnered using Landsat, questionnaire surveys, and key informant interviews. Descriptive statistics were applied to analyze LULC change and the perceptions of wetland ecosystem services and attitudes towards wetland cultivation. An ordered probit model was applied to examine the influence of household and experts’ attributes on their attitudes towards wetland cultivation. The findings indicated that despite acknowledging the direct and indirect benefits of wetlands, the majority of households and experts/ department heads have preferred transforming wetlands into farmland. Household age, asset holding size (land and livestock) and livelihood strategies (crop farming and non-farm) as well as the institutional perspectives of experts and department heads have significantly influenced the desire to convert wetlands into agricultural land. Driven by the rising young households’ interest and local governments’ goal to create jobs have led to the distribution of wetlands to unemployed graduates and landless households. Consequently, significant (48%) areas of the wetlands have been transformed into farmlands over the past 36 years. It was found that the environmental soundness of wetlands cultivation has not been considered when converting them into farmland. Efforts should be exerted to expand employment opportunities to minimize the heavy reliance of rural people on wetland farming. Besides, it is imperative to execute agro-ecological evaluation prior to converting wetlands into farmland to ensure farming decisions are environmentally responsible.*

3. The introduction does not sufficiently address both the positive and negative impacts of wetlands on agroecological systems within the study areas.

**Response**: we believe that the positive and negative impacts of wetlands on agro-ecological systems within our study areas should not be discussed in the introduction part. The reports or discussions on positive and negative impact of the study wetlands are parts of the discussion. We have made our effort to address in the discussion sections. We acknowledged the relevance of reporting or discussing the negative impact of farming on the wetland ecosystems and vice versa in the introduction part, which was not included before

4. There is also a strong possibility that wetlands are being converted into farmland due to existing environmental and socio-economic pressures.

**Response**: It is written in the introduction part and synthesized in the discussion section

5. The study lacks clearly stated objectives in the concluding paragraph of the introduction, which weakens the overall focus of the paper.

**Response:** Done based on your suggestion

6. The authors do not clearly specify the data analysis techniques or the software used.

**Response**: the software used for data analysis are reported in the revised manuscript (SPSS and STATA)

7. There is also insufficient explanation of how dependent and independent variables were analyzed. Although a framework for independent variables is presented,

**Response:** We could not see the inefficiency of the variables explanation. We believe that the dependent and dependent variables are clearly explained. Considering your concern, we have made some clarifications

8. The paper does not clearly link this framework to the results or data analysis.

**Response:** The relationships between dependent and independent variables that were hypothesized in the methodology part are analyzed and their linkage are reported in the results section

7. The results section needs substantial revision. Instead of presenting and interpreting findings, the authors continue describing methodological aspects.

**Response**: the results particularly the profiles of survey participants, which were reported in the methodology part, are moved to the results section

9. This section should focus strictly on the study findings, supported by appropriate data presentation.

**Response**: done based on the request

10. Discussion sections require significant rewriting to improve clarity, coherence, and depth of analysis.

Response: The discussion section has r-written. We hope that the re-written discussion is clear, coherent and deep

11. The discussion should critically interpret the findings in relation to existing literature,

**Response: we** believe that critical interpretation of the findings has done while modify the discussion

12. The conclusion should clearly summarize key insights and provide meaningful recommendations.

Response: similar to discussion section, modification on conclusion part has done and we hope that the modified conclusion meets your expectation

Reviewer 3

1. The abstract parts mostly focused on Excessive Focus on Negation and Insufficient Elaboration of the Key Finding Hence improved these parts from abstract.

Response: your observations regarding the abstract's limitations are completely acceptable. It is re-written. We are confident that the revisions are substantial, and we hope the updated abstract meets your expectations.

2. The study involved Field research (collecting data in a natural setting). While the focus is social science make Clarify if any specific regional/local administrative permission was obtained for field research and KIIs in the Woredas (Bure and Womberma).

Response: the data collection was conducted after getting the permission from local (woreda) administration. This is reported under ethical approval section
