# Supplementary material for: Wetland conversion to farmland in Bure and Womberma Woredas, Northwestern Ethiopia: Implications for sustainable land use
Source: PLoS One. 2026 Jul 2;21(7):e0352888. doi: 10.1371/journal.pone.0352888 (PMC13327261; doi:10.1371/journal.pone.0352888)
Supplement: Peer review history

# Peer review: Decision Letter 0

**DOI:** 10.1371/journal.pone.0352888.r001

**Authors:** Muhammad Umer Arshad

23 Nov 2025

PONE-D-25-48156

Transforming wetlands into farmland for the sake of addressing landlessness and creating employment opportunity: the case of three wetlands in Bure and Womberma Woredas, Northwestern Ethiopia

PLOS ONE

Dear Dr. Enyew,

Thank you for submitting your manuscript to PLOS ONE. After careful consideration, we feel that it has merit but does not fully meet PLOS ONE’s publication criteria as it currently stands. Therefore, we invite you to submit a revised version of the manuscript that addresses the points raised during the review process.

Please submit your revised manuscript by Jan 07 2026 11:59PM. If you will need more time than this to complete your revisions, please reply to this message or contact the journal office at plosone@plos.org. When you're ready to submit your revision, log on to https://www.editorialmanager.com/pone/ and select the 'Submissions Needing Revision' folder to locate your manuscript file.

Please include the following items when submitting your revised manuscript:

- A rebuttal letter that responds to each point raised by the academic editor and reviewer(s). You should upload this letter as a separate file labeled 'Response to Reviewers'.
- A marked-up copy of your manuscript that highlights changes made to the original version. You should upload this as a separate file labeled 'Revised Manuscript with Track Changes'.
- An unmarked version of your revised paper without tracked changes. You should upload this as a separate file labeled 'Manuscript'.

If you would like to make changes to your financial disclosure, please include your updated statement in your cover letter. Guidelines for resubmitting your figure files are available below the reviewer comments at the end of this letter.

If applicable, we recommend that you deposit your laboratory protocols in protocols.io to enhance the reproducibility of your results. Protocols.io assigns your protocol its own identifier (DOI) so that it can be cited independently in the future. For instructions see: https://journals.plos.org/plosone/s/submission-guidelines#loc-laboratory-protocols. Additionally, PLOS ONE offers an option for publishing peer-reviewed Lab Protocol articles, which describe protocols hosted on protocols.io. Read more information on sharing protocols at https://plos.org/protocols?utm_medium=editorial-email&utm_source=authorletters&utm_campaign=protocols.

We look forward to receiving your revised manuscript.

Kind regards,

Muhammad Umer Arshad

Academic Editor

PLOS ONE

Journal Requirements:

When submitting your revision, we need you to address these additional requirements.

1. Please ensure that your manuscript meets PLOS ONE's style requirements, including those for file naming. The PLOS ONE style templates can be found at

https://journals.plos.org/plosone/s/file?id=wjVg/PLOSOne_formatting_sample_main_body.pdf and

https://journals.plos.org/plosone/s/file?id=ba62/PLOSOne_formatting_sample_title_authors_affiliations.pdf

2. Please provide additional details regarding participant consent. In the ethics statement in the Methods and online submission information, please ensure that you have specified (1) whether consent was informed and (2) what type you obtained (for instance, written or verbal, and if verbal, how it was documented and witnessed). If your study included minors, state whether you obtained consent from parents or guardians. If the need for consent was waived by the ethics committee, please include this information.

If you are reporting a retrospective study of medical records or archived samples, please ensure that you have discussed whether all data were fully anonymized before you accessed them and/or whether the IRB or ethics committee waived the requirement for informed consent. If patients provided informed written consent to have data from their medical records used in research, please include this information.

3. Thank you for stating the following in the Acknowledgments Section of your manuscript:

“This research was financially supported by Blue Nile Water Institute through its thematic research project.”

We note that you have provided funding information that is not currently declared in your Funding Statement. However, funding information should not appear in the Acknowledgments section or other areas of your manuscript. We will only publish funding information present in the Funding Statement section of the online submission form.

Please remove any funding-related text from the manuscript and let us know how you would like to update your Funding Statement. Currently, your Funding Statement reads as follows:

“The author(s) received no specific funding for this work.”

Please include your amended statements within your cover letter; we will change the online submission form on your behalf.

4. Thank you for stating the following financial disclosure:

“The author(s) received no specific funding for this work.”

Please state what role the funders took in the study.  If the funders had no role, please state: "The funders had no role in study design, data collection and analysis, decision to publish, or preparation of the manuscript."

If this statement is not correct you must amend it as needed.

Please include this amended Role of Funder statement in your cover letter; we will change the online submission form on your behalf.

5. We note that your Data Availability Statement is currently as follows: “All relevant data are within the manuscript and its Supporting Information files.”

Please confirm at this time whether or not your submission contains all raw data required to replicate the results of your study. Authors must share the “minimal data set” for their submission. PLOS defines the minimal data set to consist of the data required to replicate all study findings reported in the article, as well as related metadata and methods (https://journals.plos.org/plosone/s/data-availability#loc-minimal-data-set-definition).

For example, authors should submit the following data:

- The values behind the means, standard deviations and other measures reported;

- The values used to build graphs;

- The points extracted from images for analysis.

Authors do not need to submit their entire data set if only a portion of the data was used in the reported study.

If your submission does not contain these data, please either upload them as Supporting Information files or deposit them to a stable, public repository and provide us with the relevant URLs, DOIs, or accession numbers. For a list of recommended repositories, please see https://journals.plos.org/plosone/s/recommended-repositories.

If there are ethical or legal restrictions on sharing a de-identified data set, please explain them in detail (e.g., data contain potentially sensitive information, data are owned by a third-party organization, etc.) and who has imposed them (e.g., an ethics committee). Please also provide contact information for a data access committee, ethics committee, or other institutional body to which data requests may be sent. If data are owned by a third party, please indicate how others may request data access.

6. Please ensure that you refer to Figures 5, 6, 7  and 8 in your text as, if accepted, production will need this reference to link the reader to the figure.

7. We note that Figures 1, 2, 3 and 4 in your submission contain map and satellite images which may be copyrighted. All PLOS content is published under the Creative Commons Attribution License (CC BY 4.0), which means that the manuscript, images, and Supporting Information files will be freely available online, and any third party is permitted to access, download, copy, distribute, and use these materials in any way, even commercially, with proper attribution. For these reasons, we cannot publish previously copyrighted maps or satellite images created using proprietary data, such as Google software (Google Maps, Street View, and Earth). For more information, see our copyright guidelines: http://journals.plos.org/plosone/s/licenses-and-copyright.

We require you to either (1) present written permission from the copyright holder to publish these figures specifically under the CC BY 4.0 license, or (2) remove the figures from your submission:

1. You may seek permission from the original copyright holder of Figure(s) [#] to publish the content specifically under the CC BY 4.0 license.

We recommend that you contact the original copyright holder with the Content Permission Form (http://journals.plos.org/plosone/s/file?id=7c09/content-permission-form.pdf) and the following text:

“I request permission for the open-access journal PLOS ONE to publish XXX under the Creative Commons Attribution License (CCAL) CC BY 4.0 (http://creativecommons.org/licenses/by/4.0/). Please be aware that this license allows unrestricted use and distribution, even commercially, by third parties. Please reply and provide explicit written permission to publish XXX under a CC BY license and complete the attached form.”

Please upload the completed Content Permission Form or other proof of granted permissions as an "Other" file with your submission.

In the figure caption of the copyrighted figure, please include the following text: “Reprinted from [ref] under a CC BY license, with permission from [name of publisher], original copyright [original copyright year].”

2. If you are unable to obtain permission from the original copyright holder to publish these figures under the CC BY 4.0 license or if the copyright holder’s requirements are incompatible with the CC BY 4.0 license, please either i) remove the figure or ii) supply a replacement figure that complies with the CC BY 4.0 license. Please check copyright information on all replacement figures and update the figure caption with source information. If applicable, please specify in the figure caption text when a figure is similar but not identical to the original image and is therefore for illustrative purposes only.

The following resources for replacing copyrighted map figures may be helpful:

USGS National Map Viewer (public domain): http://viewer.nationalmap.gov/viewer/

The Gateway to Astronaut Photography of Earth (public domain): http://eol.jsc.nasa.gov/sseop/clickmap/

Maps at the CIA (public domain): https://www.cia.gov/library/publications/the-world-factbook/index.html and https://www.cia.gov/library/publications/cia-maps-publications/index.html

NASA Earth Observatory (public domain): http://earthobservatory.nasa.gov/

Landsat: http://landsat.visibleearth.nasa.gov/

USGS EROS (Earth Resources Observatory and Science (EROS) Center) (public domain): http://eros.usgs.gov/#

Natural Earth (public domain): http://www.naturalearthdata.com/

8. We note you have included a table to which you do not refer in the text of your manuscript. Please ensure that you refer to Tables 5, 6 and 7 in your text; if accepted, production will need this reference to link the reader to the Table

9. If the reviewer comments include a recommendation to cite specific previously published works, please review and evaluate these publications to determine whether they are relevant and should be cited. There is no requirement to cite these works unless the editor has indicated otherwise.

Additional Editor Comments:

Why were these specific wetlands selected?

Please provide enough detail on the Landsat methodology.

How was the ordered probit model validated?

What are the quantitative outcomes for the perception variables?

The abstract also includes unsupported theoretical claims, including the rejection of Malthusian theory, Maslow’s hierarchy, and the tragedy of the commons. There is no justification for these claims.

The methods are not rigorously described.

Which Landsat sensors were used (TM, ETM+, OLI)?

What were the pre-processing steps?

Which classification method was used?

Accuracy assessment is missing.

The sampling method is unclear.

Measurement scales for perception are not clearly defined.

What reliability test was used?

The probit model is not explained well.

In the discussion, the results are overinterpreted.

There is insufficient connection between the empirical data and the theoretical claims.

[Note: HTML markup is below. Please do not edit.]

Reviewers' comments:

Reviewer's Responses to Questions

**Comments to the Author**

1. Is the manuscript technically sound, and do the data support the conclusions?

The manuscript must describe a technically sound piece of scientific research with data that supports the conclusions. Experiments must have been conducted rigorously, with appropriate controls, replication, and sample sizes. The conclusions must be drawn appropriately based on the data presented.

Reviewer #1: Partly

Reviewer #2: Yes

**********

2. Has the statistical analysis been performed appropriately and rigorously?

Reviewer #1: No

Reviewer #2: Yes

**********

3. Have the authors made all data underlying the findings in their manuscript fully available?

The PLOS Data policy requires authors to make all data underlying the findings described in their manuscript fully available without restriction, with rare exception (please refer to the Data Availability Statement in the manuscript PDF file). The data should be provided as part of the manuscript or its supporting information, or deposited to a public repository. For example, in addition to summary statistics, the data points behind means, medians and variance measures should be available. If there are restrictions on publicly sharing data—e.g. participant privacy or use of data from a third party—those must be specified.

Reviewer #1: No

Reviewer #2: Yes

**********

4. Is the manuscript presented in an intelligible fashion and written in standard English?

PLOS ONE does not copyedit accepted manuscripts, so the language in submitted articles must be clear, correct, and unambiguous. Any typographical or grammatical errors should be corrected at revision, so please note any specific errors here.

Reviewer #1: Yes

Reviewer #2: Yes

**********

5. Review Comments to the Author

Please use the space provided to explain your answers to the questions above. You may also include additional comments for the author, including concerns about dual publication, research ethics, or publication ethics. (Please upload your review as an attachment if it exceeds 20,000 characters)

Reviewer #1: The abstract parts mostly focused on Excessive Focus on Negation and Insufficient Elaboration of the Key Finding Hence improved this parts from abstract. The study involved Field research (collecting data in a natural setting). While the focus is social science make Clarify if any specific regional/local administrative permission was obtained for field research and KIIs in the Woredas (Bure and Womberma).

,

Reviewer #2: I would like to give my comment and suggestion for the manuscript

Title:

It needs to sightly modification to their `` Driving factors of wetlands into farmland for the sake of addressing landlessness and ……``

Absract:

The authors have presented a well-founded abstract; however, the structure of certain sentences could be refined to improve readability. Some sentences are overly complex or lack clear transitions between ideas, which may hinder comprehension. A careful review and restructuring of these sentences would strengthen the clarity of the abstract and provide readers with a more accessible context for the study.

Background:

The authors have presented a well-founded background however; the sequence of paragraph have been disordered. So, it needs to be reconstructed paragraph under background that will be focused: The first paragraph show that ecological and socioeconomical important of wetland in worldwide, Africa, Ethiopia and study sites.

The second paragraph will show to negative impact and community perception on the wetland

The third paragraph will consist of driving factors of wetland into foreland

The fourth paragraph will be written the effect of farmland than wetland resource or viceversal

The fifth paragraph will be showed to limitation of data, driving factors of wetland into farmland, current percentage of wetland and the action treatment of government and community participation

Materials and Methods

The authors have presented the study sites. However, it needs to seasonal variation, estimate of water volume, source of water, association linked with other dams, shedding areas GPS data, reference in 1985, 1995, 2010 and 2021. Additionally, authors will mention about estimation of human population size and grazing animals

Methods of data collection

Authors will mention the shapefiles receded year

Survey Questionnaire

It need clarify the sort out the list of question with in the categories

Wetland areas were not mentioned or data collection methods to their useful and harmful for the community

Data analysis

Authors will sort out dependent variable (Y) output and independent variables (x) factors clearly.

Results

Authors did not mention loss of plants and animals’ species and also other ecological damage when wetland converted to farmland. On other hands, these was not mentioned to avoid the community problems or remined the same or increasing to in the area

Discussion

Your discussion and conclusion were antagonistic each other in same parts but not all. So, it needs to clarification and mention based on your results

**********

6. PLOS authors have the option to publish the peer review history of their article (what does this mean?). If published, this will include your full peer review and any attached files.

If you choose “no”, your identity will remain anonymous but your review may still be made public.

**Do you want your identity to be public for this peer review?**  For information about this choice, including consent withdrawal, please see our Privacy Policy.

Reviewer #1: No

Reviewer #2: No

**********

[NOTE: If reviewer comments were submitted as an attachment file, they will be attached to this email and accessible via the submission site. Please log into your account, locate the manuscript record, and check for the action link "View Attachments". If this link does not appear, there are no attachment files.]

To ensure your figures meet our technical requirements, please review our figure guidelines: https://journals.plos.org/plosone/s/figures

You may also use PLOS’s free figure tool, NAAS, to help you prepare publication quality figures: https://journals.plos.org/plosone/s/figures#loc-tools-for-figure-preparation.

NAAS will assess whether your figures meet our technical requirements by comparing each figure against our figure specifications.

Attachment Submitted filename: My comments nd suggestion.docx

# Author comment: Author response to Decision Letter 1

**DOI:** 10.1371/journal.pone.0352888.r002

16 Jan 2026

We sincerely thank the academic editor and reviewers for your time and efforts to comment on our article and provide constructive comments to further improve its quality. We have incorporated the responses of the comments provided by the academic editor and reviewers. The responses are reported in a rebuttal letter

Attachment Submitted filename: A rebuttal letter.docx

# Peer review: Decision Letter 1

**DOI:** 10.1371/journal.pone.0352888.r003

**Authors:** Muhammad Umer Arshad, Muhammad Umer Arshad

27 Apr 2026

PONE-D-25-48156R1

Transforming wetland ecosystem into farmland in Bure and Womberma Woredas, Northwestern Ethiopia: a strategy for job creation and alleviating land shortages

PLOS One

Dear Dr. Enyew,

Thank you for submitting your manuscript to PLOS ONE. After careful consideration, we feel that it has merit but does not fully meet PLOS ONE’s publication criteria as it currently stands. Therefore, we invite you to submit a revised version of the manuscript that addresses the points raised during the review process.

Please submit your revised manuscript by Jun 11 2026 11:59PM. If you will need more time than this to complete your revisions, please reply to this message or contact the journal office at plosone@plos.org. When you're ready to submit your revision, log on to https://www.editorialmanager.com/pone/ and select the 'Submissions Needing Revision' folder to locate your manuscript file.

Please include the following items when submitting your revised manuscript:

- A letter that responds to each point raised by the academic editor and reviewer(s). You should upload this letter as a separate file labeled 'Response to Reviewers'.
- A marked-up copy of your manuscript that highlights changes made to the original version. You should upload this as a separate file labeled 'Revised Manuscript with Track Changes'.
- An unmarked version of your revised paper without tracked changes. You should upload this as a separate file labeled 'Manuscript'.

If you would like to make changes to your financial disclosure, please include your updated statement in your cover letter. Guidelines for resubmitting your figure files are available below the reviewer comments at the end of this letter.

If applicable, we recommend that you deposit your laboratory protocols in protocols.io to enhance the reproducibility of your results. Protocols.io assigns your protocol its own identifier (DOI) so that it can be cited independently in the future. For instructions see: https://journals.plos.org/plosone/s/submission-guidelines#loc-laboratory-protocols. Additionally, PLOS ONE offers an option for publishing peer-reviewed Lab Protocol articles, which describe protocols hosted on protocols.io. Read more information on sharing protocols at https://plos.org/protocols?utm_medium=editorial-email&utm_source=authorletters&utm_campaign=protocols.

As the corresponding author, your ORCID iD is verified in the submission system and will appear in the published article. PLOS supports the use of ORCID, and we encourage all coauthors to register for an ORCID iD and use it as well. Please encourage your coauthors to verify their ORCID iD within the submission system before final acceptance, as unverified ORCID iDs will not appear in the published article. *Only*  the individual author can complete the verification step; PLOS staff *cannot*  verify ORCID iDs on behalf of authors.

We look forward to receiving your revised manuscript.

Kind regards,

Muhammad Umer Arshad

Academic Editor

PLOS One

Journal Requirements:

If the reviewer comments include a recommendation to cite specific previously published works, please review and evaluate these publications to determine whether they are relevant and should be cited. There is no requirement to cite these works unless the editor has indicated otherwise.

Additional Editor Comments :

Revision has improved the paper, but it still lacks a clear statement of novelty and contribution. In the theoretical section, the inclusion of many theories makes it feel scattered; I suggest focusing on one or two key frameworks and linking them clearly for consistency. In addition, revise for simple, direct language and remove redundancy, especially in the introduction and theoretical sections, as the manuscript still needs improvement in writing clarity, grammar, and flow; many sentences are long and repetitive, making it hard to follow. Also, improve organization by tightening the background and theoretical section. The results are somewhat descriptive and would benefit from more interpretation, and the discussion should better link findings with existing literature. The conclusion is somewhat long (repetitive of the results), make it short and concise. There are also some irrelevant references; reduce and refine the reference list. Please also carefully address and incorporate the reviewer comments.

[Note: HTML markup is below. Please do not edit.]

Reviewers' comments:

Reviewer's Responses to Questions

**Comments to the Author**

1. If the authors have adequately addressed your comments raised in a previous round of review and you feel that this manuscript is now acceptable for publication, you may indicate that here to bypass the “Comments to the Author” section, enter your conflict of interest statement in the “Confidential to Editor” section, and submit your "Accept" recommendation.

Reviewer #2: All comments have been addressed

**********

2. Is the manuscript technically sound, and do the data support the conclusions?

The manuscript must describe a technically sound piece of scientific research with data that supports the conclusions. Experiments must have been conducted rigorously, with appropriate controls, replication, and sample sizes. The conclusions must be drawn appropriately based on the data presented.

Reviewer #2: (No Response)

**********

3. Has the statistical analysis been performed appropriately and rigorously?

Reviewer #2: Yes

**********

4. Have the authors made all data underlying the findings in their manuscript fully available?

The PLOS Data policy requires authors to make all data underlying the findings described in their manuscript fully available without restriction, with rare exception (please refer to the Data Availability Statement in the manuscript PDF file). The data should be provided as part of the manuscript or its supporting information, or deposited to a public repository. For example, in addition to summary statistics, the data points behind means, medians and variance measures should be available. If there are restrictions on publicly sharing data—e.g. participant privacy or use of data from a third party—those must be specified.

Reviewer #2: Yes

**********

5. Is the manuscript presented in an intelligible fashion and written in standard English?

PLOS ONE does not copyedit accepted manuscripts, so the language in submitted articles must be clear, correct, and unambiguous. Any typographical or grammatical errors should be corrected at revision, so please note any specific errors here.

Reviewer #2: Yes

**********

6. Review Comments to the Author

Please use the space provided to explain your answers to the questions above. You may also include additional comments for the author, including concerns about dual publication, research ethics, or publication ethics. (Please upload your review as an attachment if it exceeds 20,000 characters)

Reviewer #2: Title:

Here are several improved and more appealing title options that avoid the phrase “job creation and alleviating land shortages” while keeping the meaning strong and professional: Wetland Conversion to Farmland in Bure and Womberma Woredas, Northwestern Ethiopia: Implications for Sustainable Land Use. If you want it to sound more academic, more policy-focused.

Abstract:

Wetland cultivation is influenced by the characteristics of both households and experts. Due to limited farmland availability, farmers often expand cultivation into wetlands without fully considering agroecological principles. Additionally, insights from multidisciplinary experts provide important perspectives on this issue. Therefore, it is essential to incorporate agroecologists’ evaluations when assessing the conversion of wetlands into farmland, ensuring that decisions are made in line with environmental conditions.

Introduction:

The authors did not address the beneficial and adverse effects of wetlands on agroecology in the study sites. Moreover, there is a strong likelihood that wetlands may be converted into farmland due to prevailing environmental conditions. Additionally, the authors failed to clearly state the study objectives in the final paragraph of the introduction section.

Research Methods:

The authors adequately describe the research methodology. However, they fail to specify data analysis techniques or the appropriate software used to clearly distinguish dependent variables respect to independent variables analyses. In addition, that rewrite the framework of independent variables under methodology part but no writes result and data analyzed

Results:

Authors should be rewriting only the results but not describe of methodology

**********

7. PLOS authors have the option to publish the peer review history of their article (what does this mean?). If published, this will include your full peer review and any attached files.

If you choose “no”, your identity will remain anonymous but your review may still be made public.

**Do you want your identity to be public for this peer review?**  For information about this choice, including consent withdrawal, please see our Privacy Policy.

Reviewer #2: No

Reviewer #=3:

The abstract parts mostly focused on Excessive Focus on Negation and Insufficient Elaboration of the Key Finding Hence improved this parts from abstract. The study involved Field research (collecting data in a natural setting). While the focus is social science make Clarify if any specific regional/local administrative permission was obtained for field research and KIIs in the Woredas (Bure and Womberma).

**********

[NOTE: If reviewer comments were submitted as an attachment file, they will be attached to this email and accessible via the submission site. Please log into your account, locate the manuscript record, and check for the action link "View Attachments". If this link does not appear, there are no attachment files.]

To ensure your figures meet our technical requirements, please review our figure guidelines: https://journals.plos.org/plosone/s/figures

You may also use PLOS’s free figure tool, NAAS, to help you prepare publication quality figures: https://journals.plos.org/plosone/s/figures#loc-tools-for-figure-preparation.

NAAS will assess whether your figures meet our technical requirements by comparing each figure against our figure specifications.

Attachment Submitted filename: Comment and suggestion to this review paper.docx

Attachment Submitted filename: Reviewer Comment and suggestion to this paper.docx

# Author comment: Author response to Decision Letter 2

**DOI:** 10.1371/journal.pone.0352888.r004

30 May 2026

We sincerely appreciate the academic editor and reviewers for dedicating their time and providing valuable feedback to help us improve our manuscript. We have made our effort to incorporate the responses of the comments provided by academic and reviewers. The responses are reported in a rebuttal letter.

Attachment Submitted filename: Rebuttal letter second revision.docx

# Editor report: Decision Letter 2

**DOI:** 10.1371/journal.pone.0352888.r005

**Authors:** Muhammad Umer Arshad, Muhammad Umer Arshad, Muhammad Umer Arshad

16 Jun 2026

Wetland Conversion to Farmland in Bure and Womberma Woredas, Northwestern Ethiopia: Implications for Sustainable Land Use

PONE-D-25-48156R2

Dear Dr. Enyew,

We’re pleased to inform you that your manuscript has been judged scientifically suitable for publication and will be formally accepted for publication once it meets all outstanding technical requirements.

Within one week, you’ll receive an e-mail detailing the required amendments. When these have been addressed, you’ll receive a formal acceptance letter and your manuscript will be scheduled for publication.

An invoice will be generated when your article is formally accepted. Please note, if your institution has a publishing partnership with PLOS and your article meets the relevant criteria, all or part of your publication costs will be covered. Please make sure your user information is up-to-date by logging into Editorial Manager at Editorial Manager® and clicking the ‘Update My Information' link at the top of the page. For questions related to billing, please contact billing support.

If your institution or institutions have a press office, please notify them about your upcoming paper to help maximize its impact. If they’ll be preparing press materials, please inform our press team as soon as possible -- no later than 48 hours after receiving the formal acceptance. Your manuscript will remain under strict press embargo until 2 pm Eastern Time on the date of publication. For more information, please contact onepress@plos.org.

Kind regards,

Muhammad Umer Arshad

Academic Editor

PLOS One

Additional Editor Comments (optional):

The manuscript has been substantially improved through the review process, and the authors have adequately addressed the concerns raised in previous rounds of review. In its current form, the manuscript is suitable for publication and I recommend acceptance.

Reviewers' comments:

# Editor report: Acceptance letter

**DOI:** 10.1371/journal.pone.0352888.r006

**Authors:** Muhammad Umer Arshad, Muhammad Umer Arshad, Muhammad Umer Arshad

PONE-D-25-48156R2

PLOS One

Dear Dr. Getnet Eneyew,

I'm pleased to inform you that your manuscript has been deemed suitable for publication in PLOS One. Congratulations! Your manuscript is now being handed over to our production team.

At this stage, our production department will prepare your paper for publication. This includes ensuring the following:

* All references, tables, and figures are properly cited

* All relevant supporting information is included in the manuscript submission,

* There are no issues that prevent the paper from being properly typeset

You will receive further instructions from the production team, including instructions on how to review your proof when it is ready. Please keep in mind that we are working through a large volume of accepted articles, so please give us a few days to review your paper and let you know the next and final steps.

Lastly, if your institution or institutions have a press office, please let them know about your upcoming paper now to help maximize its impact. If they'll be preparing press materials, please inform our press team within the next 48 hours. Your manuscript will remain under strict press embargo until 2 pm Eastern Time on the date of publication. For more information, please contact onepress@plos.org.

You will receive an invoice from PLOS for your publication fee after your manuscript has reached the completed accept phase. If you receive an email requesting payment before acceptance or for any other service, this may be a phishing scheme. Learn how to identify phishing emails and protect your accounts at https://explore.plos.org/phishing.

If we can help with anything else, please email us at customercare@plos.org.

Thank you for submitting your work to PLOS ONE and supporting open access.

Kind regards,

PLOS ONE Editorial Office Staff

on behalf of

Dr. Muhammad Umer Arshad

Academic Editor

PLOS One
